# Modelling Mitochondrial Disease in Human Pluripotent Stem Cells: What Have We Learned?

**DOI:** 10.3390/ijms22147730

**Published:** 2021-07-20

**Authors:** Cameron L. McKnight, Yau Chung Low, David A. Elliott, David R. Thorburn, Ann E. Frazier

**Affiliations:** 1 Murdoch Children’s Research Institute, Royal Children’s Hospital, Parkville, VIC 3052, Australia; cameron.mcknight@mcri.edu.au (C.L.M.); yau.low@mcri.edu.au (Y.C.L.); david.elliott@mcri.edu.au (D.A.E.); david.thorburn@mcri.edu.au (D.R.T.); 2 Department of Paediatrics, University of Melbourne, Parkville, VIC 3052, Australia; 3 Victorian Clinical Genetics Services, Royal Children’s Hospital, Parkville, VIC 3052, Australia

**Keywords:** stem cell, hPSC, iPSC, hESC, CRISPR-Cas9, mtDNA, disease modelling, mitochondrial disease

## Abstract

Mitochondrial diseases disrupt cellular energy production and are among the most complex group of inherited genetic disorders. Affecting approximately 1 in 5000 live births, they are both clinically and genetically heterogeneous, and can be highly tissue specific, but most often affect cell types with high energy demands in the brain, heart, and kidneys. There are currently no clinically validated treatment options available, despite several agents showing therapeutic promise. However, modelling these disorders is challenging as many non-human models of mitochondrial disease do not completely recapitulate human phenotypes for known disease genes. Additionally, access to disease-relevant cell or tissue types from patients is often limited. To overcome these difficulties, many groups have turned to human pluripotent stem cells (hPSCs) to model mitochondrial disease for both nuclear-DNA (nDNA) and mitochondrial-DNA (mtDNA) contexts. Leveraging the capacity of hPSCs to differentiate into clinically relevant cell types, these models permit both detailed investigation of cellular pathomechanisms and validation of promising treatment options. Here we catalogue hPSC models of mitochondrial disease that have been generated to date, summarise approaches and key outcomes of phenotypic profiling using these models, and discuss key criteria to guide future investigations using hPSC models of mitochondrial disease.

## 1. Introduction

So much more than just “the powerhouse of the cell”, mitochondria also handle critically important biochemical processes including cell signalling, iron-sulfur (Fe/S) cluster biogenesis, apoptosis, and calcium homeostasis [1,2]. Nonetheless, mitochondrial diseases are classified as disorders of energy generation that either directly or indirectly affect ATP production via the oxidative phosphorylation (OXPHOS) system. One of the most complex groups of inherited genetic conditions, they can result from mutations in either nuclear (nDNA) or mitochondrial DNA (mtDNA). Additionally, patient phenotypes can be highly heterogeneous and tissue specific, making them difficult to diagnose and study [3].

Despite there being over 1100 known mitochondrial proteins, only 37 genes are encoded on the small (16 kb) circular double-stranded mtDNA (2 ribosomal RNAs (rRNAs), 22 transfer RNAs (tRNAs), and 13 OXPHOS subunits) [4,5]. Unlike the nuclear genome, mtDNA is entirely maternally inherited [6] and each cell contains hundreds to thousands of copies [7]. Copy number varies between cell types, as can the proportion of mutated copies [8,9,10]. The ratio of mutant to wild type mtDNA (i.e., heteroplasmy) that leads to disease can be both cell type and mutation specific [11,12]. Heteroplasmy can be affected by a number of factors, including a genetic bottle neck during embryogenesis [13] and genetic drift over generations [14]. The OXPHOS system itself is made up of five multi-subunit complexes (Complexes I-V; CI-CV) inserted into the inner mitochondrial membrane [15]. With only 13 of the ~90 OXPHOS subunits being mtDNA encoded [16], mitochondria are dependent on import of more than 250 additional nuclear encoded proteins and assembly factors required for OXPHOS function [17,18].

With over 330 different mitochondrial disease genes identified between the two genomes, it has become apparent that it will be a monumental task to model all of the distinct genes and mutations [19,20]. Furthermore, available patient cell lines such as fibroblasts do not always display disease phenotypes [21], and accessing disease relevant tissues from patients can be difficult or limited. These efforts have been further compounded by the inability of some non-human model systems to recapitulate the human phenotypes for certain key disease genes [22]. In the case of *SURF1*-related Leigh syndrome for example, *SURF1* knockout mice and pigs did not show significant neurological phenotypes despite their characteristic decreased complex IV expression, with the mice also living longer and showing resistance to cytotoxic stress [23,24,25]. A range of other mitochondrial disease-specific mouse models have been developed over the years, but many of them still have caveats that make it challenging to conduct treatment studies [26].

Despite several promising treatment options, there are no clinically validated treatments for mitochondrial diseases [3,27], with most patients instead receiving tailored symptomatic treatments, even following a genetic diagnosis [28,29]. With a relative lack of non-human models amenable to high-throughput screening approaches, human pluripotent stem cell (hPSC) models of mitochondrial disease offer a promising alternative as a drug discovery platform [3].

## 2. Pluripotent Stem Cells in Mitochondrial Disease Modelling

**Figure 1:**
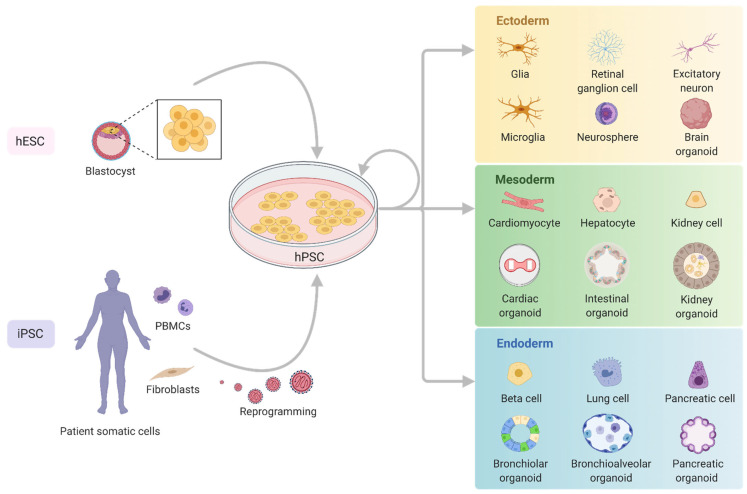
Generation and utility of human stem cell models. Human pluripotent stem cells (hPSCs) can be generated either from the inner cell mass of a blastocyst, known as human embryonic stem cells (hESCs), or by reprogramming a host’s somatic cells, most commonly fibroblasts or peripheral blood mononuclear cells (PBMCs), to make induced pluripotent stem cells (iPSCs). Both forms of hPSCs are capable of indefinite self-renewal and can differentiate to the three primary germ layers. This capacity to differentiate can be directed experimentally to form terminally differentiated cell types or more complex organoid models, making hPSCs suitable for modelling the multitude of systems affected by mitochondrial diseases.

Pluripotent stem cells are defined by the capacity to give rise to cells from all three germ layers and indefinitely self-renew (Figure 1) [30]. This unique potential is advantageous for human mitochondrial disease modelling.

Since the first human embryonic stem cells (hESCs) were isolated from the inner cell mass of an excess IVF blastocyst in 1998, it was clear that these cells had great potential for disease modelling and cell therapy [31]. However, over the past 15 years, much research has gone into optimising induced pluripotent stem cell (iPSC) technologies to ensure human somatic cells reprogrammed to a stem cell fate are equivalent to hESC in every relevant metric [32,33]. Now considered largely equivalent, it is acceptable to choose either embryonic or induced human pluripotent stem cells (hPSCs) for disease modelling based on experimental requirements. Nonetheless, caveats remain for iPSCs, including variations in methylation state, copy number, and transcription that can be influenced by reprogramming methods and somatic cell origin [34].

Numerous protocols have been developed to differentiate hPSCs to specific cell fates either using growth factors and small molecules to mimic signals from embryogenesis for a given cell lineage, or via direct gene regulation approaches [35,36]. This can be done in 2D to look at more homogeneous cell populations, or in 3D as an organoid system to better approximate the tissue environment for a given cell type. While organoids offer a more complex cell network with multiple cell types, they can be difficult to form consistently and can require significant optimisation for different cell lines [37,38].

In a mitochondrial disease context, hPSCs have many attractive characteristics. Principally, hPSCs are highly glycolytic and are not heavily reliant on OXPHOS for energy generation [39]. This implies that mitochondrial mutant cell lines should be stable and grow normally in a stem cell state. However, there could be complications when differentiating some more severe mitochondrial disease models since differentiation often requires rapid mitochondrial biogenesis and increased reliance on OXPHOS as the cells mature [40,41,42,43]. These issues would likely be mutation specific and may require modification of the differentiation conditions to better support the mutant line [44,45,46,47,48]. However, not all mutations will have differentiation issues and the resulting cell types can overcome the tissue specific challenges of studying these complex disorders.

This review will highlight hPSC models of mitochondrial disease that have been generated to date for the purposes of disease modelling and has not included any lines generated for other purposes (e.g., studying cell therapies [49,50], diabetes [51], aging [52]). We have also opted not to include genes linked to other metabolic pathways (e.g., mitochondrial fatty acid oxidation) or mitochondrial-linked disorders with distinct phenotypes such as Parkinson’s disease [53,54,55,56,57]. We will summarise approaches used to phenotype and investigate these models as well as key outcomes. We will also suggest common validation criteria that could better standardise future studies of hPSC models of mitochondrial disease and any downstream investigations.

## 3. Generation of Human Pluripotent Stem Cell Mitochondrial Disease Models

### 3.1. Technologies and Considerations for Generating hPSC Disease Models

**Figure 2:**
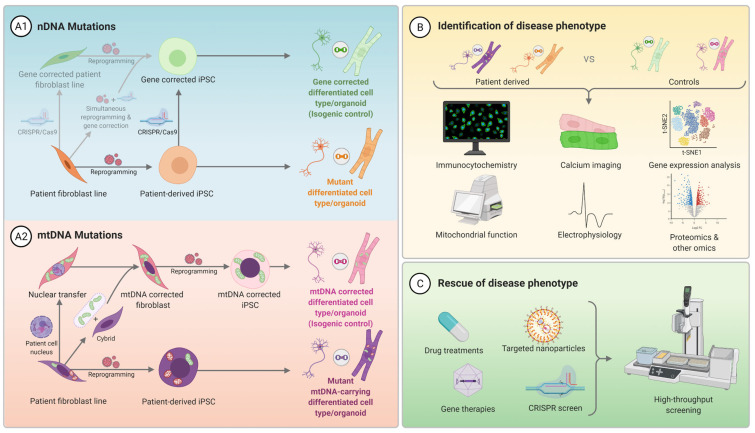
Pathways to modelling mitochondrial diseases using iPSCs. (**A1**) Using genetic editing approaches (e.g., CRISPR-Cas9), gene corrected iPSCs can be generated for individual mitochondrial disease patient fibroblast lines (or PBMCs) with nDNA mutations. For the studies included in this review, the generation of isogenic controls by gene editing has all been done post-reprogramming, but it is also possible to do simultaneously, or prior to reprogramming. Gene editing has also been done inversely in hPSCs to generate mutation lines with the parental line as the isogenic control. (**A2**) Generation of isogenic controls for mtDNA mutation lines is commonly achieved prior to reprogramming, either by patient cell nuclear transfer to an enucleated WT mtDNA host cell line or by cybrid formation. (**B**) Differentiation of controls alongside hPSC mutant lines makes it possible to identify phenotypic differences in both nDNA and mtDNA models using different functional analyses and/or omics technologies. (**C**) By adapting functional analysis techniques for high-throughput screens, it is possible to identify or validate candidate treatment options such as compounds, targeted nanoparticle approaches, gene therapies, or CRISPR-Cas9 screens to pinpoint target pathways.

Generation of a mitochondrial disease specific hPSC model is not dissimilar to any other hPSC line when it comes to nDNA mutations. However, modelling mtDNA mutations involves some unique challenges and techniques that must be considered due to potential issues with mtDNA heteroplasmy. For an overview of the pathways available for generation of hPSC models and considerations for mitochondrial disease, see Figure 2(A1,A2), and sections below.

An important consideration for mitochondrial disease or other hPSC studies is the choice of controls to be used for comparison. Many of the publications reported here have directly compared patient iPSC lines to a pool of non-isogenic controls, i.e., with a different genetic background (and not always age and/or sex matched). Although a pool of at least three age- and sex-matched non-isogenic controls may be a reasonable approach, due to the variabilities in reprogramming and differentiation processes it has been shown that only isogenic controls can provide sufficiently robust data to detect subtle phenotypic differences [58,59]. The need for just a single control may also be important for scalability of downstream drug screens, making this an important consideration during cell line generation [59]. Fortunately, generation of isogenic controls has become increasingly simple as gene editing becomes more commonplace. Using CRISPR-Cas9 to correct nDNA patient mutations can be done before [60], after [61], or even during reprogramming [62] (Figure 2(A1)). For mtDNA disorders, this can be accomplished with traditional techniques like somatic cell nuclear transfer or cybrids [63,64,65]. Both approaches result in the patient nDNA in a wild type mtDNA background but must be done prior to reprogramming (Figure 2(A2)). Additionally, in some cases, the reprogramming process can result in some clones with wild type homoplasmy, which can be used as isogenic controls for mtDNA-based stem cell studies [21,45,66].

#### 3.1.1. Reprogramming of Somatic Cells into iPSCs

**Figure 3:**
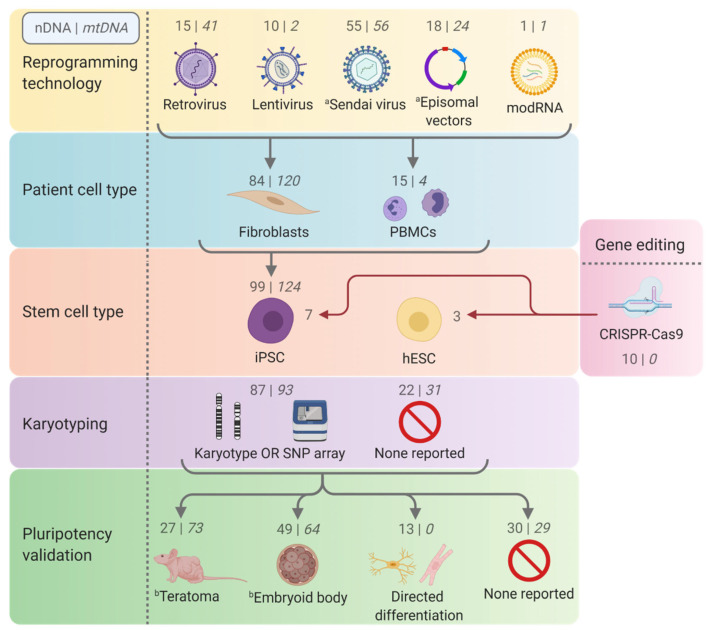
Generation and validation of mitochondrial disease iPSC lines. A graphical illustration of the various pathways used to generate and validate the mitochondrial disease hPSC lines described in Table A1 and Table A2, indicating the number of nDNA and mtDNA clones generated from each somatic cell type by the technologies used in the process. The counts include all validated and reported clones generated for each nDNA and mtDNA cell line (with >30% mutant heteroplasmy for mtDNA lines) to highlight the discrepancy in the number of clones often generated and screened for mtDNA lines versus nDNA lines due to variable heteroplasmy following reprogramming. For some nDNA lines, CRISPR-Cas9 gene editing was used to generate mitochondrial disease gene mutations in wild type hPSC backgrounds. Two key steps in the validation of iPSC lines are karyotyping or SNP (single nucleotide polymorphism) array analysis and pluripotency validation. Karyotype analysis minimises the risk that any unanticipated chromosomal rearrangements may have occurred during reprogramming. Pluripotency validation must demonstrate the ability to generate the three germ layers, in addition to the expression of pluripotency markers (e.g., SSEA4, TRA-1-81, TRA-1-61) [75]. ^a^ Fibroblasts were used as the patient somatic cell type for all lines except for 10 nDNA and 4 mtDNA mutation clones using Sendai virus and 5 nDNA mutation clones reprogrammed using episomal vectors. ^b^ 10 nDNA and 42 mtDNA lines were double counted as they were validated by both teratoma and embryoid body assays.

Reprogramming to an iPSC fate involves reverting a terminally differentiated cell type, typically fibroblasts or peripheral blood mononuclear cells (PBMCs), to an uncommitted pluripotent stem cell fate by transient expression of Oct4 (Pou5f1), Sox2, Klf4, and c-Myc [67]. This can be accomplished using a range of technologies and Figure 3 outlines the technologies used to generate the mitochondrial stem cell models published to date [68].

Since the first extensive characterisations of iPSC mitochondrial disease models in 2013 [21,45,69], the choice of reprogramming method has largely been influenced by the predominant technology at the time of generation [68]. Integrative viral vectors like retrovirus and lentivirus were common in early iPSC studies due to their high efficiency and ease of use. However, they were later revealed to be subpar activators of endogenous pluripotency genes and carried a high risk of detrimental gene integration events [70]. This led the field to move towards non-integrative viral approaches like Sendai virus. Although significantly lower in efficiency [71], Sendai virus is still a popular reprogramming method for its relative simplicity and cost effectiveness. To date, Sendai virus has been used to reprogram most mitochondrial disease iPSC models (Figure 3). More recent advances in reprogramming technology have come in the form of non-integrative non-viral systems, such as episomal vectors and modified RNA (modRNA) [72,73]. Each advance in reprogramming technology has offered increased safety and efficacy for therapeutic applications of iPSCs and these trickle down as new standards for research use as efficiency increases and costs decrease over time. Although non-integrative non-viral technologies were only used to generate a small subset of the mitochondrial disease iPSC models included here (Figure 3), episomal vectors are already beginning to phase out Sendai virus in the therapeutic setting and will likely account for most mitochondrial disease iPSC lines generated in the future [74].

In the context of mitochondrial disease, genetic background can play a major role, making iPSCs a particularly valuable modelling tool. For patients with complex nDNA mutations or variants of uncertain significance (VUS), where there may be secondary mutations contributing to the phenotype, this could be the only approach to effectively capture the genetic complexity of these conditions.

#### 3.1.2. Gene Editing

For both iPSCs or hESCs, CRISPR-Cas9 is an invaluable tool in the generation of either patient corrected isogenic controls or recreating a patient mutation in a wild type genetic background [76] (Figure 2(A1,A2)), and has been successfully used to generate several mitochondrial disease hPSC models (Figure 3 and Table A1). CRISPR-Cas9 technology has been adapted from the bacterial viral defence system to work efficiently as a precise genetic editing tool in human cells by generating targeted double- or single-stranded breaks within a ~20 bp guide sequence that corresponds to a single location in the nuclear genome [77].

Manipulation of the mitochondrial genome is less straightforward. Due to the apparent lack of a mitochondrial RNA import machinery, CRISPR-Cas9 does not appear to be a viable option for editing mtDNA [78]. However, other options include mitochondrial-targeted transcription activator-like effector nucleases (mitoTALENs) [79], which target specific point mutations to decrease heteroplasmy levels or even generate isogenic controls with homoplasmic WT mtDNA [79,80]. While this technique provides a powerful tool to modify heteroplasmy levels in hPSCs, it has only been applied in two of the studies identified in this review [80,81].

Other approaches, such as mitochondrial-targeted zinc-finger nucleases (mtZFN) and restriction endonucleases (mitoRE), could also produce a similar effect [82], but their use has not yet been reported in hPSCs. Similarly, the recently described RNA-free DddA-derived cytosine base editors (DdCBEs) could provide more precise control over heteroplasmy without impacting mtDNA copy number [83], but have yet to be validated in hPSCs.

### 3.2. Quality Control and Characterisation of Pluripotent Stem Cell Disease Models

The process of generating a hPSC line is lengthy and can be stressful for the cell, potentially allowing for the accumulation of deleterious genomic and phenotypic changes along the way [84]. Therefore, it is important that quality checks are performed on each cell line to ensure any phenotypes are not merely a consequence of the generation process. Several standards have been set out for therapeutic use of stem cells and the research field has adopted a number of these validation steps to ensure the quality of research cell lines [75,85]. We would therefore recommend that all mitochondrial disease hPSC models are screened for mycoplasma, normal karyotype, normal cell morphology, as well as pluripotency by FACS and trilineage differentiation to an EB, teratoma, or via directed differentiation to the three germ layers [85]. Although most of the cell lines included in this review did achieve this level of validation, there were a significant proportion that did not disclose the karyotype results of their lines and/or validation of their pluripotency (Figure 3; Table A1 and Table A2). These validation steps should be standard for all iPSC lines, not just those modelling mitochondrial diseases.

In addition to the above standard screening procedures, it is recommended that iPSCs should also undergo DNA-fingerprinting analysis. This can be done by SNP analysis of parental somatic cell DNA for cross-referencing. This was not disclosed by many of the included studies (Table A1 and Table A2) but is now considered an important step to ensure the validity of all comparisons. It is possible for cell lines to get mixed up and/or contaminated during reprogramming, or for large numbers of SNPs to change during the reprogramming process, so high-resolution DNA-fingerprinting is critical to ensure valid isogenic controls and disease models [86].

**Figure 4:**
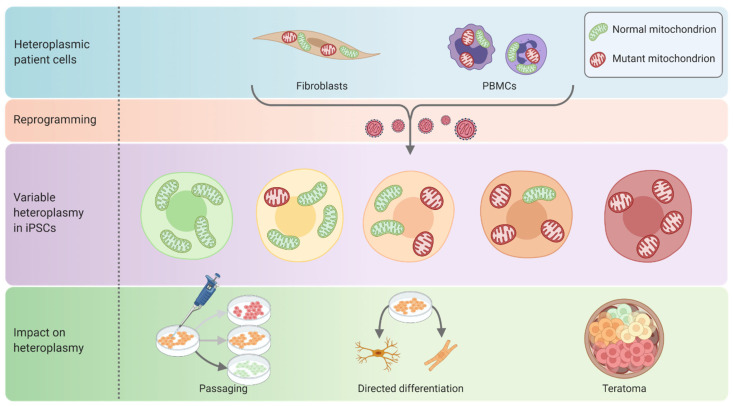
Maintenance of mtDNA heteroplasmy in iPSCs. Generation of iPSC lines from patient cells with mtDNA mutations can result in a shift in heteroplasmy from the somatic cell levels in either direction [21,66], although biased toward loss of mutant load [45,89]. Similarly, heteroplasmy can shift in either direction as heteroplasmic iPSC lines (orange cells) are passaged, with a general bias towards a decreased mutant load over time (green cells, lower mutant load; red cells, higher mutant load) [45,69,87,90]. During directed differentiation, heteroplasmy is typically unchanged from the level observed in the undifferentiated iPSCs [21,91,92]. However, during teratoma assays, different cell lineages can end up manifesting cell-type specific OXPHOS defects that may depend on the specific mtDNA mutation [21,93]. These biases appear to be selective, with mechanisms such as genetic bottleneck and genetic drift only playing a minor role [91].

mtDNA heteroplasmy in iPSCs has proven to be a challenging area that could significantly contribute to the variability found between iPSC lines and to differentiation issues (Figure 4) [87,88]. The reprogramming process itself can evidently be subject to a genetic bottleneck effect and yield hPSC clones with different levels of heteroplasmy from the same patient somatic cell line [21,66]. In general, heteroplasmy appears to bias toward a decreased mutant load following either reprogramming or extended cell culture [45,87,89], potentially indicating that high mutant loads are not well tolerated [69,90]. In contrast, directed differentiations have been reported to maintain the heteroplasmy level of the starting hPSCs [21,91,92]. While undirected differentiations in teratomas have been reported to increase the overall mutant load [93], for other mtDNA mutations, heteroplasmy levels in teratomas were unchanged from the starting iPSC population [21]. Therefore, any biases may depend on the specific mtDNA mutation and be controlled by selection, with other mechanisms such as genetic bottleneck and genetic drift only playing a minor role following reprogramming [91]. Hence, it is critical that heteroplasmy is carefully monitored and cell lines are not kept in extended culture without consistent validation of heteroplasmy. Additionally, threshold levels for the mutant load required to manifest a disease phenotype can vary considerably for each individual mtDNA mutation and need to be considered [3].

## 4. Disease Modelling

Mitochondrial disease hPSC models provide a system to study disease gene- or mutation-related pathomechanisms in tissues relevant to the clinical phenotype. Ultimately, the long-term goal of these models would be to identify a phenotype in a clinically relevant cell type that could be used to validate efficacy of targeted treatments, or for use in high-throughput treatment trials [94,95,96] (Figure 2).

There are now a wide range of endpoints that have been validated in terminally differentiated cell types to investigate the underlying cellular mechanisms of disease and efficiently identify targetable pathways. Many of these approaches can also be adapted to suit different cell types and even organoids at scale. The tissue specific nature of mitochondrial diseases means that mitochondrial function post-differentiation can be distinct to that from the undifferentiated stem cells or original fibroblast line, often greatly exaggerating any underlying defects [97]. Additionally, detailed transcriptomic and proteomic analyses can elucidate cellular compensation mechanisms and potential target pathways to inform downstream treatment studies [98,99]. Other approaches include microscopic visualization of key cellular features to determine a mutation’s impact on cell structure or function [100]. For cardiomyocytes and neurons, electrophysiology can provide highly sensitive data to identify even subtle functional changes [101]. Calcium imaging can be particularly informative in the context of mitochondrial diseases, since calcium handling is a key role of mitochondria [102,103].

## 5. Functional Studies

Only two hESC models of mitochondrial diseases have been published to date [104,105]. The remaining hPSC lines we have included are either: (i) iPSCs reprogrammed from patient somatic cells, or (ii) control iPSC lines genetically edited to possess a mutation in a relevant mitochondrial disease gene (see Table A1 and Table A2).

For the purposes of this review, we will highlight a selection of studies that have utilised hPSCs to generate clinically relevant cell types for investigation of tissue specific defects of mitochondrial diseases or for the screening of potential therapeutic treatments for mitochondrial diseases. We have focused on mitochondrial diseases for which multiple hPSC cell lines have been reported, with similar findings uncovered from the various publications. We have elected to exclude the extensive studies involving hPSC models of Friedreich’s ataxia, caused by GAA triplet-repeat expansions in *FXN*, which have been reviewed elsewhere [106]. See Table A3 and Table A4 for details regarding cell lines, controls, and functional outcomes from these hPSC-derived mitochondrial disease models, and others not specifically featured below. The studies selected used either isogenic controls or a pool of at least 3 non-isogenic controls (see Section 3.1 and Section 3.2 for recommended guidelines), and/or involved multiple publications with complementary outcomes. Understandably, modelling of mitochondrial disease using hPSCs is still in its infancy and not all reported studies will meet these criteria.

### 5.1. Barth Syndrome—TAFAZZIN

Barth syndrome (OMIM# 302060) is caused by mutations in *TAFAZZIN*. Tafazzin is responsible for the formation of mature cardiolipin (tetralinoleoyl cardiolipin; CL), an essential lipid found mainly in the mitochondrial inner membrane that stabilises multiple protein complexes for optimal function and mitochondrial health [107,108].

As cardiomyopathy is the primary cause of death, several Barth syndrome iPSC models to investigate pathomechanisms in iPSC-derived cardiomyocytes (iPSC-CMs) have been developed [109,110]. As in patient tissues, an immature cardiolipin isoform (monolysocardiolipin; MLCL) dominates in the patient iPSC-CMs, resulting in an imbalance of the MLCL:CL ratio [107,109,111]. Like previous studies in other Barth syndrome cellular models, the iPSC-CMs displayed structural remodelling of OXPHOS complexes, including recapitulation of a cardiac specific decrease in OXPHOS complex II (CII) observed in mouse models [110]. Due to these OXPHOS defects, the maximal oxygen consumption rate (OCR) in Barth syndrome iPSC-CMs was lower than controls, however, the basal respiration rate was increased [103,109,110]. This counterintuitive observation was suggested to be a result of H^+^ leak across the inner membrane, and increased F_1_F_0_ ATP synthase (complex V)-linked oxygen consumption, albeit generating ATP inefficiently [109]. As well, cardiac cells preferentially utilise fatty acids as their substrate of choice for ATP production, but retain the flexibility to use other substrates when available or compelled [112]. Due to the mitochondrial dysfunction, Barth syndrome iPSC-CMs undergo metabolic alterations, utilise less palmitate, and rely more on glycolysis for energy generation [113]. At the single cell level, quantification of sarcomeric regularity in Barth syndrome iPSC-CMs revealed sarcomeric disorganisation [109,110], although this was resolved when cardiomyocytes were engineered to align in 3D [103]. Nevertheless, Barth syndrome iPSC-CMs possessed contractility defects compared to controls following electrical stimulation in both a 2D thin muscular film format [109] and in a 3D engineered heart tissue assay [103].

It is widely recognised that elevated levels of reactive oxygen species (ROS) resulting in lipid peroxidation are a likely contributor to the cardiomyopathy observed in Barth syndrome [114]. Notably, the mitochondrial targeting ROS scavenger MitoTEMPO was shown to reduce ROS levels and improve contractility defects of Barth syndrome iPSC-CMs [103,109]. In addition, elevated ROS in Barth syndrome iPSC-CMs caused excessive activation of CaMKII, and downstream CaMKII-mediated phosphorylation of the ryanodine receptor (RYR2) at Ser2814 [103]. This led to increased diastolic Ca^2+^ leak across the sarcoplasmic reticulum, contributing to calcium homeostasis abnormalities and the contractility defects observed in Barth syndrome iPSC-CMs [103]. With encouraging results emerging from a recent clinical trial of Barth syndrome patients with elamipretide, these hPSC-CM models could prove useful for further validation studies of its therapeutic potential [115].

### 5.2. DOA and Parkinson’s Disease—OPA1

Autosomal dominant heterozygous mutations in *OPA1* (OMIM# 605290) result in optic atrophy (DOA) [116,117]. OPA1 plays a prominent role in mitochondrial membrane fusion and is essential for cellular differentiation [118]. While *OPA1* mutant iPSC-derived neural stem cells (iPSC-NSCs) and neural progenitor cells (iPSC-NPCs) can be generated relatively normally [104,119], it is unsurprising that all reported *OPA1* mutant hPSC lines encountered differentiation defects and/or increased cell death in the later stages of neural and retinal ganglion cell differentiation [104,110,119,120]. Additionally, reduced mitochondrial respiration and OXPHOS complex I (CI) related ATP synthesis defects were also common features of *OPA1* mutant iPSC-derived dopaminergic neurons (iPSC-DANs) [119,120].

Like patient somatic cells [121], *OPA1* mutant hPSCs displayed an increased accumulation of ROS and sensitivity to cell death, even at the pluripotent stage [104,110]. The addition of apoptosis- or necrosis-inhibiting factors in the early stages of differentiation were shown to be beneficial in improving the survival of neurons and avoiding neurodegeneration following prolonged culture [119].

In severe cases, neurodegeneration due to *OPA1* mutations has been reported to contribute to Parkinson’s disease [122]. A direct comparison between *OPA1* DOA versus Parkinson’s disease iPSC-DANs derived from related patients with the same mutations showed that increased mitochondrial fragmentation and cell death likely contributed to the more severe symptoms in the Parkinsonism model [120]. A microfluidic nigrostriatal pathway (a brain structure composed primarily of dopaminergic neurons) on-a-chip technique, consisting of iPSC-DANs and striatal medium spiny neurons, was developed to study functional synapse connections in the *OPA1* Parkinson’s disease model. It identified a progressive loss of dopaminergic neuron synaptic terminals due to decreased mitochondrial content and motility along the axons [123].

### 5.3. PEO and Alpers Syndrome—POLG

Mutations in *POLG* (OMIM# 174763) are responsible for an array of mitochondrial disorders with neurological manifestations, including progressive external ophthalmoplegia (PEO) [124]. The effects of compound heterozygous *POLG* mutations resulting in autosomal recessive PEO were analysed using patient derived iPSC-NSCs [125] and iPSC-DANs [126]. Consistent with the prominent role played by POLG in mtDNA replication, mutant iPSC-NSCs and iPSC-DANs displayed reduced mtDNA copy number and CI subunit expression, features of which were seen in neurons isolated from the patient brain tissues, but not iPSCs or fibroblasts [125,126]. Consequently, a reduction in the NAD^+^:NADH ratio was identified in *POLG* patient iPSC-NSCs compared to controls, while the undifferentiated patient iPSCs trended towards an increased NAD^+^:NADH ratio [125].

With disruptions in OXPHOS being touted as a major contributor to ROS production [127], *POLG* patient-derived iPSC-NSCs and iPSC-DANs showed increased ROS levels that could be improved following N-acetylcysteine amide supplementation [125,126]. An increase in ROS was otherwise not detected in the patient iPSCs, while patient fibroblasts showed reduced ROS levels instead when compared to controls [125]. Reduced ATP levels were also seen in patient iPSCs, iPSC-NSCs, and fibroblasts, but not iPSC-DANs [125,126]. Such differences between cell types highlight the importance of utilising iPSC-derived clinically relevant cell types to replicate tissue specific defects for the purposes of therapeutic investigations.

In addition to neurological presentations, autosomal recessive mutations in *POLG* can lead to Alpers syndrome (OMIM# 203700), characterized by liver failure, seizures, and neuronal degeneration [128]. Valproic acid (VA) is a common treatment for seizure disorders, however numerous patients with *POLG* mutations reportedly suffer from severe liver toxicity following VA administration. Hepatocyte-like cells generated from Alpers syndrome patient iPSCs (iPSC-Hep) displayed various mitochondrial defects (see Table A3) and were more prone to VA-induced apoptosis caused by increased ROS release due to the opening of the mitochondrial permeability transition pore (mPTP) [129]. This could be prevented by administering the mPTP inhibitor cyclosporine A, or the antioxidants carnitine and N-acetylcysteine (NAC) [129].

### 5.4. mtDNA Depletion Syndromes—DGUOK and RRM2B

*DGUOK*, encoding mitochondrial deoxyguanosine kinase, is involved in mtDNA nucleotide synthesis. Biallelic mutations in this gene can cause mtDNA depletion syndrome 3 (MTDPS3; OMIM# 251880), a condition associated with neurologic abnormalities and liver failure [130]. To identify prospective treatments for MTDPS3, *DGUOK*-deficient iPSC-Hep were generated from CRISPR-Cas9 gene-edited iPSCs [131]. In a separate study, *DGUOK* defects were investigated in patient derived iPSC-Hep and hepatocyte organoid models [132]. Compared to isogenic controls, *DGUOK^−/−^* iPSC-Hep displayed both reduced mtDNA copy number and lower expression of mtDNA encoded genes [131,132]. Mutant cells also displayed impaired mitochondrial respiration, including a significant reduction in basal OCR, maximal OCR, and ATP levels compared to controls, while extracellular lactate production (e.g., glycolysis) increased [131]. Furthermore, *DGUOK^−/−^* iPSC-Hep showed increased susceptibility to iron overload-induced ferroptosis that could be rescued either by silencing of nuclear receptor co-activator 4 (NCOA4) or treatment with NAC [132].

A library of 2400 drugs was screened for increased cellular ATP levels in *DGUOK^−/−^* iPSC-Hep to identify prospective treatments. From this screen, 15 drugs were identified to increase ATP levels by more than 20% [131]. One drug in particular, nicotinamide adenine dinucleotide (NAD), not only improved ATP production, but also consistently increased the expression of the mtDNA encoded OXPHOS genes *MT-ATP8* (CV), *MT-CO1* (CIV), *MT-CYB* (CIII), and *MT-ND1* (CI). Furthermore, treatment with NAD restored mitochondrial morphology in *DGUOK^−/−^* iPSC-Hep, increased mitochondrial membrane potential and improved OXPHOS respiration to levels comparable to WT cells [131]. NAD treatment was shown to upregulate mitochondrial biogenesis through PGC1α activation, as opposed to direct upregulation of mtDNA copy number [131,133,134]. The use of NAD in combination with other ATP elevating drug candidates acting on different pathways enhanced the improvement in ATP levels over NAD alone [131]. Notably, the authors demonstrated that NAD treatment has therapeutic potential for other mtDNA depletion syndromes, showing improved ATP levels in a *RRM2B^−/−^* iPSC-Hep model generated by CRISPR-Cas9 gene editing [131]. *RRM2B* (OMIM# 604712) encodes a subunit of the mitochondrial ribonucleotide reductase complex that catalyses the conversion of ribonucleoside diphosphates into deoxyribonucleoside diphosphates, and *RRM2B* mutations lead to MTDPS8A/8B with clinical features including a range of neurological symptoms and liver involvement.

### 5.5. Leigh Syndrome

Leigh syndrome (OMIM# 256000), a progressive neurodegenerative disorder, is the most common paediatric onset mitochondrial disease. Mutations in more than 75 genes (both nDNA and mtDNA encoded) can result in Leigh syndrome [135]. Thus far, investigations have been reported for Leigh syndrome iPSC-derived neuronal cell types possessing mutations in: *MT-ATP6* [98,136], *MT-ND5* [102], *NDUFS4* and *SURF1* [137], and *SCO2* [99].

#### 5.5.1. Complex IV Assembly Factors—SURF1 and SCO2

Deficiency of OXPHOS complex IV (cytochrome *c* oxidase; COX) accounts for approximately 15% of all Leigh syndrome diagnoses [138,139,140], with mutations in *SURF1* being the most commonly reported [135]. There are three distinct intermediates formed during CIV assembly, with several assembly factors stabilising and supporting the process, including SURF1 and SCO2. SURF1 is part of the first assembly intermediate, the MITRAC (mitochondrial translation regulation assembly intermediate of cytochrome *c* oxidase), in the inner mitochondrial membrane [141]. Mutations in *SURF1* severely reduce the levels of fully assembled CIV and result in accumulation of these assembly intermediates [142,143]. In contrast, loss of SCO2 results in degradation of mtDNA encoded subunits (COXI and II) [144]. A metallochaperone, SCO2 aids the insertion of copper into COXII, supporting formation of the MT-CO2 module [145]. While mutations in *SCO2* result in similarly decreased CIV biogenesis and have also been associated with Leigh(-like) syndrome [146,147], patients more commonly present with hypertrophic cardiomyopathy [144,148,149,150].

Inak et al. (2021) generated several *SURF1* iPSC models of CIV-linked Leigh syndrome [99]. These included both patient derived iPSC lines (one with corresponding isogenic control), as well as CRISPR-Cas9 gene-edited lines homozygous for one of the patient mutations, derived from a healthy iPSC control (Table A1). By modelling the same *SURF1* mutation in two different genetic backgrounds, any compensatory effects resulting from the patients nuclear or mitochondrial makeup could be investigated [151]. Neural differentiation of the *SURF1*-iPSC lines revealed that loss of SURF1 negatively impacts both commitment and function as early as the neural progenitor stage. iPSC-NPCs exhibited decreased neurite branching, neurite length, maximal respiration, and ATP-linked respiration. Similar defects were observed in iPSC-NPCs derived from two *NDUFS4^−/−^* iPSC lines (OXPHOS complex I subunit; Table A1), supporting the decreased branching and neurite length as a broader Leigh syndrome phenotype rather than being gene-specific [99]. Further differentiation of the *SURF1* lines to iPSC-derived neurons (iPSC-DNs; primarily dopaminergic) exacerbated these defects and showed decreased spiking and postsynaptic activity compared to isogenic controls. In a 3D neural organoid system, loss of SURF1 resulted in small poorly organised structures with significantly fewer TUJ1^+^, MAP^+^, and SYP^+^ neurons by day 90. Single cell transcriptomic analyses of *SURF1* iPSC-DNs and neural organoids indicated an imbalance between proliferation and differentiation that disrupts normal neuronal maturation [99].

A range of mitochondrial targeted treatment strategies to improve OXPHOS function were trialled in the *SURF1* iPSC-NPCs and -DNs with varying levels of success [99]. Hypoxia, which was shown to be beneficial in mouse models of *NDUFS4^−/−^* Leigh syndrome [152,153,154], resulted in increased glycolysis and exacerbated the neuronal outgrowth phenotypes in the *SURF1* neurons. While metabolic manipulations (e.g., increased glucose and pyruvate supplementation) and treatment with ROS-scavengers (NAC or alpha-tocotrienol; AT3; EPI-743) marginally reduced glycolysis, they failed to improve cell morphology or mitochondrial function. Transcriptomic data from the *SURF1* iPSC-DNs and neural organoids suggested expression of *PPARGC1A* was significantly reduced, therefore treatment with bezafibrate was trialled [99], which has previously been shown to activate peroxisome proliferator-activated receptor (PPAR) and pharmacologically upregulate PGC1α driven mitochondrial biogenesis in iPSC-NPCs [155]. Treating the *SURF1* iPSC-NPCs with 400 µM bezafibrate resulted in increased mtDNA copy number, reduced expression of pluripotency and proliferation markers, and improved neuronal outgrowth and morphology (Table A3) [99].

Crucially, bezafibrate treatment allowed the *SURF1*-neurons to undergo the metabolic switch from glycolysis to OXPHOS and nearly ameliorated all mitochondrial functional defects, supporting bezafibrate as a possible therapeutic option for *SURF1*-related Leigh syndrome [3,99]. However, it is unclear if its effectiveness depends on the timing of the treatment [155], and a recent study on a cohort of six patients with *MT-TL1* (m.3243A > G) mutations causing mitochondrial myopathy showed bezafibrate treatment was minimally effective at increasing mitochondrial biogenesis in patient skeletal muscle [156], and instead led to dose-dependent increases in mitochondrial disease biomarkers FGF-21 and GDF-15 [156,157,158]. Therefore, mitochondrial biogenesis may not be effective for all mitochondrial diseases and will require further investigations to resolve when during development it could have the most impact.

For *SCO2*, two iPSC lines were generated along with three healthy non-isogenic controls (Table A1), although most of the functional analyses only included one of these control lines [137]. Since mutations in *SCO2* are predominantly associated with cardiomyopathy, functional studies of *SCO2* iPSCs have so far been limited to iPSC-CMs. Both *SCO2* mutant lines displayed arrhythmic contractility resulting from significant underlying calcium handling defects, supported by their attenuated response to ionotropic interventions (isoproterenol, angiotensin-II, and increased extracellular calcium) and delayed recovery following caffeine exposure. Nonetheless, significant progressive mitochondrial ultrastructural defects were only observed in the *SCO2*^G193S^ mutant. It was proposed that the underlying defects in OXPHOS lead to an ATP deficit [149,159], which affects storage and handling of calcium by the sarcoplasmic reticulum and cardiomyocyte contractility [137]. Therefore, treatments targeting mitochondrial function or biogenesis could improve ATP production to support normal contractility in *SCO2* mutant iPSC-CMs [3,160]. The benefits of bezafibrate treatment seen in the *SURF1* hPSC models support this as a treatment option for *SCO2* iPSC-CMs. Likewise, differentiation of these CIV deficiency hPSC models into similar cell types (e.g., cardiomyocytes or neurons) could provide insight into shared pathogenic pathways.

#### 5.5.2. MT-ATP6

Several mtDNA mutations affecting *MT-ATP6*, a mtDNA encoded OXPHOS complex V subunit, have been associated with Leigh syndrome. Models reported so far include patient iPSCs possessing a homoplasmic m.9185T > C mutation differentiated into NPCs [98], and studies of homoplasmic m.8993T > G mutations in iPSC-DNs [136]. For both models, comparison to an array of non-isogenic controls was used.

In addition to reduced ATP production [98,136], both mutant iPSC-NPCs and iPSC-DNs displayed an increase in mitochondrial membrane potential (MMP) [98,136]. This mitochondrial hyperpolarization was not observed in *MT-ATP6* m.9185T > C mutant fibroblasts nor cybrid lines [98], although it was reported previously in *MT-ATP6* m.8993T > G cybrid models [161,162,163,164]. While the m.8993T > G iPSC-NPCs were able to compensate for reduced ATP levels through glycolysis, terminally differentiated m.8993T > G iPSC-DNs are incapable of doing so, likely due to a lack of hexokinase and lactate dehydrogenase enzymes, further exacerbating the ATP defect observed in these cells [136].

Global proteomic and transcriptomic analyses revealed that genes involved in calcium signalling and homeostasis were downregulated in mutant m.9185T > C iPSC-NPCs. As expected, these changes were not detected in mutant fibroblasts, nor the undifferentiated patient iPSCs [98]. Calcium imaging of m.9185T > C iPSC-NPCs revealed impaired calcium homeostasis. A reduction in calcium-induced calcium release following stimulation with glutamate, as well as reduced mitochondrial calcium release following MMP depolarization, was also observed [98].

Taking advantage of the increased MMP phenotype observed in the m.9185T > C NPCs, 130 FDA-approved drugs were tested to identify compounds that would ameliorate this defect [98]. This screen identified avanafil as a compound that resulted in partial depolarization of the MMP. However, avanafil treatment did not improve ATP production, nor calcium-induced cytosolic calcium release. Nevertheless, overnight treatment with avanafil did improve mitochondrial calcium release in mutant NPCs and neurons upon MMP depolarization [98]. In the m.8993T > G iPSC-DANs, targeted treatment with rapamycin improved ATP production, reduced aberrant AMP-activated protein kinase activation, and decreased susceptibility to glutamate overdose toxicity [136].

### 5.6. LHON

Leber hereditary optic neuropathy (LHON; OMIM# 535000) is a mitochondrial disorder typically caused by homoplasmic mtDNA complex I subunit mutations.

Initial studies in a LHON iPSC model carrying homoplasmic double mtDNA mutations in *MT-ND1* and *MT-ND6* determined that their differentiation efficiency to retinal ganglion cells (iPSC-RGCs) was unaffected, but apoptosis was more prominent in these cells when compared to both a non-isogenic control and a cybrid corrected isogenic control [64]. Furthermore, in LHON patient iPSC-RGCs carrying a m.11778G > A *MT-ND4* mutation, optic vesicles derived from the LHON iPSCs were smaller, with a notable difference in the appearance of the neuroblastic layer compared to controls [101]. Additionally, LHON iPSC-RGCs possessed shorter neurites, and formed fewer connections with neighbouring RGC bundles, while mitochondrial motility was also impaired [165]. NAC treatment was able to reduce ROS and improve mitochondrial motility and survival of m.11778G > A LHON iPSC-RGCs [166]. However, it is unclear if the differentiation defects were a direct manifestation of mitochondrial dysfunction (Table A4) or are linked to the increased apoptosis [64,166]. Additionally, the use of non-isogenic controls makes it difficult to assign phenotypes directly to the m.11778G > A *MT-ND4* variant [101,165]. Nonetheless, glutamate uptake was compromised in m.11778G > A LHON iPSC-RGCs. Changes in the expression levels and binding of α-amino-3-hydroxy-5-methylisoxazole-4-propionic acid (AMPA) receptor and downstream scaffold proteins involved in glutaminergic synapse signalling were detected in mutant iPSC-RGCs, both in the presence and absence of glutamate stimulation [101].

### 5.7. MELAS—MT-TL1

Mutations in the mtDNA *MT-TL1* gene, which encodes tRNA-leucine, account for a large proportion of patients with mitochondrial encephalomyopathy, lactic acidosis, and stroke-like episodes (MELAS; OMIM# 540000) [167].

In initial studies, iPSCs were generated possessing high *MT-TL1* m.3243A > G mutation levels (~80%), with mitoTALENs used to eliminate the mutant mtDNA, thereby generating an isogenic control (see Section 3.1.2) [80]. Compared to the isogenic control, mutant iPSC-NPCs displayed respiration defects (Table A4), as well as differentiation abnormalities in the later stages of neuronal differentiation after NPC formation [80,97]. The cells failed to differentiate into motor neurons, while spinal cord organoids appeared to possess shorter neurite outgrowths [168]. This was shown to result from aberrant hyperactive Notch signalling, likely a direct consequence of the m.3243A > G mutation, as general CI inhibition by rotenone treatment also induced similar Notch signalling abnormalities [168]. The differentiation and morphological defects were rescued following quenching of Notch signalling using the inhibitor DAPT [168]. In similar studies, cortical excitatory neurons with variable heteroplasmy of the *MT-TL1* m.3243A > G mutation were generated from patient iPSCs [97]. Cells with WT homoplasmy were used as an isogenic control, in addition to non-isogenic control lines. Neurons with high m.3243A > G heteroplasmy levels (>65%) displayed similar mitochondrial respiration defects to those observed in iPSC-NPCs [97]. Morphologically, mutant neurons were shorter, and possessed fewer branch points and synaptic terminals at the single cell level. Mitochondrial content along the neuronal axons was also lower compared to controls. Interestingly, in m.3243A > G iPSC-neurons with a high mutant load (>80%), CI appeared to be actively sequestered into autophagosomes and cleared by mitophagy during further neuronal differentiation, contributing to CI deficiency in these neurons [21].

At the network level, spontaneous neuronal activity recorded by multi-electrode array indicated that neurons with high m.3243A > G heteroplasmy levels had abnormal electrophysiological properties: reduced mean firing rate, reduced network burst rates, and increased random spike events outside of network bursts compared to controls [97]. In comparison, neurons with intermediate heteroplasmy levels (~30%) were relatively normal, indicative of the threshold effect for mtDNA mutations.

To specifically investigate the pathomechanisms causing stroke-like episodes in MELAS [169], MELAS-iPSCs (generated by [80]) were differentiated into endothelial cells (iPSC-ECs) and compared against the mitoTALEN corrected isogenic control [48]. In addition to reduced differentiation efficiency and poor endothelial tube formation, m.3243A > G iPSC-ECs were found to be pro-atherogenic and pro-inflammatory, expressing high levels of ROS and consequently increased oxidation of low-density lipoprotein (LDL). Mutant cells expressed increased levels of ‘pro-adhesive’ VCAM-1 isoform b, priming them for an inflammatory response and suggesting MELAS may have an atherosclerosis-like pathology. Even unstimulated, these iPSC-ECs showed more than 2-fold greater monocyte adhesion and the adhered monocytes expressed increased IL-8, setting the stage for proinflammatory niche formation [48]. Supporting the potential of hPSC models for preclinical identification of promising therapeutic options, treatment of MELAS iPSC-ECs with antioxidants CoQ10, Vitamin C, and edaravone (FDA approved for treatment of amyotrophic lateral sclerosis [170]), improved endothelial tube formation, as well as reduced ROS and inflammation levels [48].

## 6. Conclusions

It is clear from the publications highlighted here that hPSC models of mitochondrial disease recapitulate key aspects of human disease phenotypes in vitro and provide tissue specific insights into disease pathomechanisms. However, the validation approaches employed need to be sufficiently robust to detect any inconsistencies between the mutant and controls resulting from the hPSC generation process [75,85]. Additionally, the importance of isogenic controls cannot be overstated when assigning pathogenicity to a given genetic variant, and it is entirely possible that subtle changes could be overlooked with pooled controls [59]. From the studies we have identified, 57% of the cell lines reported (114 in total) provided complete validation information including pluripotency, differentiation potential, and karyotype, with 40% of all iPSC lines (112 in total) also including lineage validation. Furthermore, of the 61 separate cell lines (54% of total) that underwent functional analyses, only 18% exclusively used isogenic controls, while a further 23% included isogenic controls alongside non-isogenic, and 25% made comparisons against at least 3 pooled non-isogenic controls.

For mitochondrial diseases in particular, variation in heteroplasmy poses a significant challenge for mtDNA hPSC models [87], but is manageable for the purposes of treatment studies. Heteroplasmy often appears to remain stable following directed differentiation [45,87,91]. Therefore, it is practical to check heteroplasmy levels before and after differentiation to ensure they are within the phenotypic range for patients with the same mutation [45,171,172]. Additionally, use of mitochondrial targeted editing strategies like mitoTALENs and DdCBEs could help achieve a greater level of control prior to differentiation [80,81,83].

The stem cell field is continuously improving differentiation protocols to generate increasingly mature cell types [173,174,175] and models that are more disease relevant (i.e., organoids) [176]. Basic differentiation protocols typically only produce cells with gene expression profiles equivalent to embryonic cells [177]. These systems often require some form of secondary enrichment to yield a pure population of the relevant cells [178], and/or maturation steps (either supplemental, environmental, mechanical, or a combination) to achieve a disease relevant developmental stage [179,180]. For example, some neuronal cell types may benefit from co-culture with glial cells to achieve functional maturity [181,182]. Cell maturity significantly impacts on mitochondrial content and morphology [160,183]. Therefore, it is important to consider the suitability of a differentiation and maturation technique for any desired endpoints. Although organoid models may be more relevant, they may limit throughput due to added complexity of the culture format and a limited number of compatible assays [37,184]. They can also add additional complexity when it comes to dosing, penetration, and consistency between replicates due to the presence of multiple cell types in varied proportions [185,186]. Therefore, studies may require a mixture of 2D and 3D models depending on the required throughput and the disease relevant cell types.

Overall, mitochondrial disease hPSC models remain a promising option for mechanistic and therapeutic investigations, with a large number of models already generated having significant untapped potential (69 nDNA/27 different genes: 45 mtDNA/10 different genes and 4 large deletions) (Table A1 and Table A2). However, some of the preliminary functional analyses from these models have failed to provide any disease specific pathomechanistic insights above those that could be, or have already been, observed with other more common human cellular models (e.g., fibroblasts; Table A3 and Table A4). It is also evident that there are some fundamental roadblocks in progressing from cell line generation to the therapeutic testing stages of these disease models, as only two publications (1 nDNA: 1 mtDNA) performed large scale intervention screens on differentiated cells [98,131]. Several studies (9 nDNA: 4 mtDNA) were able to perform more targeted screens using fewer than 6 compounds, with many only trialling a single compound after identifying a key pathway (Table A3 and Table A4). This is indicative of the challenges associated with obtaining sufficient differentiated cells for screening, maintaining the purity of the target cell population, and achieving the highest possible maturity for a given cell type [187]. Another key hurdle to drug screens is the need for functional endpoints that provide sufficiently robust disease-relevant phenotypes to draw conclusive results [188]. Transcriptomics and other “omics” approaches, particularly in combination (i.e., multi-omics), have the potential to provide a more complete picture of where and how processes are being regulated in a disease state [189]. These studies could aid pathway identification in differentiated cell types to inform downstream drug screens and elucidate disease specific cellular mechanisms, with some mitochondrial disease hPSC models reporting these data already (Table A3 and Table A4) [48,65,98,99,104,119,190,191]. Despite the hurdles to screening, feasibility has been shown in some model systems and these approaches could be adapted to other terminally differentiated cell types [98,131]. Given the pace of hPSC development, it is likely that these hPSC models will be increasingly deployed to investigate tissue specific disease mechanisms and to screen for promising candidate drug treatments for mitochondrial disease. For mitochondrial diseases, where the clinical and genetic heterogeneity means there will likely be no one-size-fits-all treatment, these models have great potential to elucidate a range of treatment approaches suitable for downstream clinical trials.

## Appendix A

Table A1 (nDNA models) and Table A2 (mtDNA models) are intended as a resource summarising hPSC models of mitochondrial disease that have been generated to date for the purposes of disease modelling (i.e., directly or secondarily affecting OXPHOS function). Not included are lines generated for other purposes (e.g., studying cell therapies, diabetes, aging), or those generated involving genes linked to other metabolic pathways or mitochondrial-linked disorders with specific phenotypes. We have attempted to compile a comprehensive list but acknowledge we may have overlooked some relevant hPSC models. For many of the included lines, isogenic control iPSC or parental iPSC lines may have been specifically generated or occurred incidentally (e.g., low/no mutant heteroplasmy), but these are not listed in this table. For mtDNA lines with multiple clones generated, all clones subjected to some form of validation (e.g., pluripotency, etc) with >30% mtDNA mutant load have been included, noting however that the mutant threshold required to manifest a biochemical defect can vary widely for each individual mtDNA mutation. For some of the reported mtDNA models, the pathogenicity of the reported variants is uncertain, as indicated. We have not included the cell line generated in [192] and the reported variant m.4216T > C in [65], due to available population data (MITOMAP and gnomAD 3.1) that indicates the mtDNA variants are of questionable pathogenicity (i.e., high population frequency and/or mtDNA haplotype marker).

**Table A1:** nDNA models.

Gene	Disease	Cell Line ID	Mutation ^a^	Cell Line Origin ^b^	Gene Editing /Reprogramming	Pluripotency	Trilineage Potential	Karyotype; Lineage Validation ^c^	Mycoplasma Check	Ref.
*AARS2*	COXPD8	LUMCi024-A	p.[(R958*)]; [(R592W);(V730M)]	M; 4 days; Fib	Sendai virus	Morphology; IF; FACS	EB differentiation	G-banding; Yes	Yes	[193]
*AARS2*	COXPD8	LUMCi025-A	p.[(R958*)]; [(R592W);(V730M)]	M; 1 day; Fib	Sendai virus	Morphology; IF; FACS	EB differentiation	G-banding; Yes	Yes	[193]
*ACO2*	DOA	IISHDOi006-A	p.[(E667K)];[=]	M; 30 yr; Fib	Sendai virus	Morphology; AP; qPCR; IF	EB differentiation	G-banding; Yes	Yes	[194]
*AIFM1*	AN	CPGHi003-A	p.[(R422Q)];[0]	M; 49 yr; PBMC	Episomal vectors	Morphology; RT-qPCR; IF	EB differentiation	G-banding; Yes	Yes	[195]
*ATAD3A*	HSP	HEL142 (2 clones)	p.[(G355D)];[=]	F; 35 yr; Fib	Episomal vectors	qPCR; IF	N/D	N/D	N/D	[196]
*C1QBP*	COXPD	XACHi010-A	p.[(L275F)]; [(L275F)]	M; 14 yr; PBMC	Sendai virus	Morphology; IF; FACS	Trilineage differentiation	G-banding; Yes	Yes	[197]
*COQ2*	MSA-C	MSA_A (3 clones)	p.[(R387*)]; [(V393A)]	M; 61 yr; PBMC	Episomal vectors	Morphology; IF	Teratoma	G-banding; N/D	N/D	[198]
*COQ4*	CoQ10 deficiency	CQ4-Ipsc (4 clones)	p.[(E161D)];[=]	F; 4 yr; Fib	Sendai virus	Morphology; AP; RT-qPCR; IF; FACS; hypomethylation	Teratoma	G-banding; Yes	Yes	[199,200]
*COX6A2*	CIV deficiency	WAe009-A-47	p.[A16Lfs*18]; [A16Lfs*18]	F; hESC-WA09	CRISPR-Cas9 induced mutation	Morphology; RT-qPCR; IF; FACS	Teratoma	G-banding; Yes	Yes	[105]
*DGUOK*	MTDPS3	DGUOK^Δ14/Δ5^ iPSC	p.[W166*]; [H167Lfs*213]	M; iPS-SV20	CRISPR-Cas9 induced mutation	N/D	N/D	N/D	N/D	[131]
*DGUOK*	MTDPS	Patient 1	p.[F256*];[F256*]	F; 2 mo; Fib	Retrovirus	Morphology	N/D	G-banding; N/D	N/D	[132]
*DGUOK*	MTDPS	Patient 2	[p.A2S; c.591G > A]; [c.142 + 1G > A],	M; 2 mo; Fib	Retrovirus	Morphology	N/D	G-banding; N/D	N/D	[132]
*DNAJC19*	DCMA	Patient 1	c.[130-1G > C]; [130-1G > C]	F; 1.5 yr; PBMC	Sendai virus	IF	N/D	SNP microarray; N/D	N/D	[201]
*DNAJC19*	DCMA	Patient 2	c.[130-1G > C]; [130-1G > C]	M; 11 yr; PBMC	Sendai virus	IF	N/D	SNP microarray; N/D	N/D	[201]
*DNAJC19*	DCMA	LIBUCi001-A	c.[130-1G > C]; [130-1G > C]	M; 8 yr; Fib	Sendai virus	Morphology; IF; FACS	EB differentiation	G-banding; Yes	Yes	[202]
*DNAJC19*	DCMA	LIBUCi002-A	c.[130-1G > C]; [130-1G > C]	F; 10 yr; Fib	Sendai virus	Morphology; IF; FACS	EB differentiation	G-banding; Yes	Yes	[202]
*DNAJC19*	DCMA	JMUi001-A	p.[(A44Vfs*12)];[(S46Rfs*3)]	M; iPS- JMUi001-A.	CRISPR-Cas9 induced mutation	Morphology; IF; FACS	EB differentiation	G-banding; Yes	Yes	[202]
*ECHS1*	Leigh-like syndrome	UOMi001-A	p.[(A172V)]; [(K284Pfs*7)]	M; 13 yr; PBMC	Sendai virus	Morphology; qPCR; IF	EB differentiation	KaryoStat analysis; Yes	Yes	[203]
*FBXL4*	MTDPS13	SHCDNi001-A	p.[(L332Tfs*3)];[(L332Tfs*3)]	F; 1 yr; PBMC;	Sendai virus	Morphology; IF; FACS	Trilineage differentiation	G-banding; Yes	Yes	[204]
*GDAP1*	CMT2K	CMT2-FiPS4F1	p.[(Q163*)]; [(T288Nfs*3)]	M; 45 yr; Fib	Sendai virus	Morphology; qPCR; IF	EB differentiation	G-banding; Yes	N/D	[205]
*GFM1*	Mitochondrial encephalopathy	GFM1SV.25	p.[G469Vfs*84];[R671C]	F; 5.5 yr; Fib	Sendai virus	Morphology; AP; qPCR; IF; hypomethylation	EB differentiation	G-banding; Yes	N/D	[206]
*MFN2*	CMT2A	Patient 1 (3 clones)	p.[(R364W)];[=]	M; 42 yr; Fib	Retrovirus	Morphology; qPCR; IF	EB differentiation	G-banding; Yes	N/D	[207]
*MFN2*	CMT2A	CMT2A-1 (3 clones)	p.[(A383V)];[=]	F; 7 yr; Fib	Episomal vectors	Morphology; RT-PCR; IF	Yes (data not shown)	N/D	N/D	[191]
*MFN2*	CMT2A	CMT2A-2 (3 clones)	p.[(A383V)];[=]	F; 12 yr; Fib	Episomal vectors	Morphology; RT-qPCR; IF	Yes (data not shown)	N/D	N/D	[191]
*MFN2*	CMT2A	ZJUCHi002-A	p.[(P251L)];[=]	M; 8 yr; Urine cells	Retrovirus	Morphology; AP; qPCR; IF	EB differentiation	G-banding; Yes	Yes	[208]
*MFN2*	MSL	JUCTCi012-A	p.[(R707W)]; [(R707W)]	F; 39 yr; Fib	Sendai virus	Morphology; IF; FACS	EB differentiation	G-banding; Yes	Yes	[209]
*NDUFS4*	Leigh syndrome	NDU_1	p.[(K154fs)];[(K154fs)]	M; 5 mo; Fib	Sendai virus	N/D	N/D	SNP microarray; Yes	Yes	[99]
*NDUFS4*	Leigh syndrome	NDU_2	p.[(R106*)]; [(R106*)]	F; 4 mo; Fib	Sendai virus	N/D	N/D	SNP microarray; Yes	Yes	[99]
*NDUFV1*	Leigh syndrome	UOMi002-A	p.[(Y177Lfs*2)];[(E214K)]	F; 2.5 yr; PBMC	Sendai virus	Morphology; IF	EB differentiation	hPSC Genetic Analysis Kit; Yes	Yes	[210]
*OPA1*	DOA	VO-iPSC	c.[(2496+1G > T)];[=]	Fib	Retrovirus/ Sendai virus	IF	Teratoma	N/D	N/D	[211]
*OPA1*	DOA	OL-iPSC	c.[(2496+1G > T)];[=]	Fib	Retrovirus/ Sendai virus	IF	Teratoma	N/D	N/D	[211]
*OPA1*	DOA ‘plus’	Oex2054SV.4	p.[(Q621*)];[=]	M; Fib	Sendai virus	Morphology; AP; qPCR; IF; hypomethylation	EB differentiation	G-banding; Yes	N/D	[212]
*OPA1*	Behr syndrome	iPS-OPA1-BEHR	c.[610+364G>A];p.[I437M]	F; 48 yr; Fib	Episomal vectors	AP; RT-qPCR; IF	EB differentiation	SNP microarray; Yes	N/D	[213]
*OPA1*	DOA ‘plus’	IISHDOi003-A	p.[(S545R)];[=]	M; 43 yr; Fib	Sendai virus	Morphology; AP; qPCR; IF	EB differentiation	G-banding; Yes	Yes	[214]
*OPA1*	Parkinson’s disease	PD-OPA1 G488R (2 clones)	p.[G488R];[=]	M; 74 yr; Fib	Sendai virus	Morphology; qPCR; IF	Trilineage differentiation	G-banding; N/D	N/D	[119]
*OPA1*	Parkinson’s disease	PD-OPA1 A495V #72	p.[A495V[;[=]	M; 70 yr; Fib	Sendai virus	Morphology; qPCR; IF	Trilineage differentiation	G-banding; N/D	N/D	[119]
*OPA1*	Parkinson’s disease	Opa1P (2 clones)	c.[(33-34ins9)];[=]	M; 62 yr; Fib	Sendai virus	Morphology; Pluritest; FACS	N/D	SNP microarray; Yes	Yes	[120]
*OPA1*	DOA	Opa1 (2 clones)	c.[(33-34ins9)];[=]	F; 84 yr; Fib	Sendai virus	Morphology; Pluritest; FACS	N/D	SNP microarray; Yes	Yes	[120]
*OPA1*	DOA	OPA1+/− hESCs (2 clones)	N/D (haploinsufficiency)	F; hESC-WA22	CRISPR-Cas9 induced mutation	IF	N/D	SNP microarray; N/D	N/D	[104]
*OPA1*	DOA	OPA1+/- iPSC 1	p.[(V958Gfs*3)];[=]	M; PBMC	Sendai virus	N/D	N/D	N/D	N/D	[104]
*OPA1*	DOA	OPA1+/- iPSC2	p.[(V958Gfs*3)];[=]	M; PBMC	Sendai virus	N/D	N/D	N/D	N/D	[104]
*OPA1*	DOA	BIOi002-A	c.[2708_ 2711delTTAG];[=]	M; 27 yr; PBMC	Episomal vectors	Morphology; RT-PCR; FACS	Teratoma	G-banding; Yes	Yes	[215]
*PDK3*	CMTX6	iPSCCMTX6	p.[(R158H)];[0]	M; Fib	Episomal vectors	RT-qPCR; IF	N/D	G-banding; Yes	N/D	[216]
*PMPCB*	Leigh-like syndrome	DII-2 iPSC (3 clones)	p.[I422T];[I422T]	M; Fib	Episomal vectors	IF; PluriTest	N/D	G-banding; N/D	Yes	[217]
*POLG*	Alpers syndrome	AHS iPS 1	p.[A467T];c.[1251-2A > T]	M; 3.5 yr; Fib	Retrovirus	Morphology; AP; RT-qPCR; IF	Teratoma; EB differentiation	G-banding; N/D	N/D	[129]
*POLG*	Alpers syndrome	AHS iPS 2	p.[A467T];c.[3626_3629dup]	F; 2 yr; Fib	Retrovirus	AP; Morphology; RT-qPCR; IF	Teratoma; EB differentiation	G-banding; N/D	N/D	[129]
*POLG*	N/D	PG64SV.2	p.[(W748S)]; [(W748S)]	F; 36 yr; Fib	Sendai virus	Morphology; AP; qPCR; IF; hypomethylation	EB differentiation	G-banding; Yes	N/D	[218]
*POLG*	PEO and Parkinson’s disease	CSC-35 (3 clones)	p.[(Q811R)];[=]	F; 24 yr; Fib	Sendai virus	Morphology; AP; IF	EB differentiation	G-banding; Yes	N/D	[190]
*POLG*	PEO	WS5A (3 clones)	p.[W748S];[W748S]	F; Fib	Retrovirus	Morphology; RT-qPCR; IF; FACS;	Hep, CM, and neuronal differentiation	G-banding; N/D	Yes	[125,126]
*POLG*	PEO	CP2A (2 clones)	p.[A467T]; [W748S]	M; Fib	Retrovirus	Morphology; RT-qPCR; IF; FACS	Hep, CM, and neuronal differentiation	G-banding; N/D	Yes	[125,126]
*RRM2B*	MTDPS8A/B	RRM2B^−/−^ iPSC	p.[R36Sfs*55]; [R36Sfs*55]	M; iPS-SV20	CRISPR-Cas9 induced mutation	N/D	N/D	N/D	N/D	[131]
*SAMHD1*	AGS	PEIi002 (3 clones)	homozygous exon 14 and 15 deletion	M; PBMC	Sendai virus	Morphology; RT-qPCR; IF	Trilineage differentiation	CGH-array; Yes	Yes	[219]
*SAMHD1*	AGS	hSAMHD1-R290H+Q548X	p.[(R290H)];[(Q548*)]	M; PBMC	Sendai virus	FACS	EB differentiation	G-banding; Yes	N/D	[220]
*SCO2*	CIV deficiency	SCO2G193S	p.[(G193S)]; [(G193S)]	M; 4 mo; Fib	Lentivirus	IF	Teratoma	G-banding; N/D	N/D	[137]
*SCO2*	CIV deficiency	SCO2E140K	p.[(E140K)]; c.[(17ins(19))] ^d^	M; 13 wk; Fib	Lentivirus	IF	Teratoma	G-banding; N/D	N/D	[137]
*SURF1*	Leigh syndrome	SURF1_Mut: S1	p.[(V177G)]; [(V177G)]	M; 9 yr; Fib	Sendai virus	RT-PCR; IF	EB differentiation	SNP microarray and G-banding; Yes	Yes	[99]
*SURF1*	Leigh syndrome	SURF1_Mut: S2	p.[(G257R)]; [(G257R)]	M; 20 mo; Fib	Sendai virus	RT-PCR; IF	EB differentiation	SNP microarray, G-banding and WGS; Yes	Yes	[99]
*SURF1*	Leigh syndrome	C1_Mut (2 clones)	p.[(G257R)]; [(G257R)]	F; iPS-XM001	CRISPR-Cas9 induced mutation	N/D	N/D	SNP microarray; Yes	Yes	[99]
*TAZ*	Barth syndrome	TAZ10 (2 clones)	p.[G197V];[0]	M; Fib	Lentivirus	Morphology; AP; RT-PCR; IF; hypomethylation	Teratoma; EB differentiation	N/D	N/D	[221]
*TAZ*	Barth syndrome	TAZ13 (3 clones)	c.[110-1G > C];[0]	M; Fib	Lentivirus	Morphology; AP; RT-PCR; IF; hypomethylation	Teratoma; EB differentiation	N/D	N/D	[221]
*TAZ*	Barth syndrome	TAZ15 (3 clones)	p.[R57L];[0]	M; Fib	Lentivirus	Morphology; AP; RT-PCR; IF; hypomethylation	Teratoma; EB differentiation	N/D	N/D	[221]
*TAZ*	Barth syndrome	BTH-H	p.[(D173Tfs*12)];[0]	M; Fib	Retrovirus	Morphology; RT-qPCR; IF	Teratoma	G-banding; N/D	N/D	[109]
*TAZ*	Barth syndrome	BTH-C	p.[(S110P)];[0]	M; Fib	Modified RNA	Morphology; RT-qPCR; IF	Teratoma	G-banding; N/D	N/D	[109]
*TAZ*	Barth syndrome	PGP1-TAZ^c.517delG^	p.[(D173Tfs*12)];[0]	M; iPS-PGP1	CRISPR-Cas9 induced mutation	Morphology; RT-qPCR; IF	Teratoma	G-banding; N/D	N/D	[109]
*TAZ*	Barth syndrome	PGP1-TAZ^c.517ins^	p.[(D173Efs)];[0]	M; iPS-PGP1	CRISPR-Cas9 induced mutation	Morphology; RT-qPCR; IF	Teratoma	G-banding; N/D	N/D	[109]
*TAZ*	Barth syndrome	WMUi002-A	p.[(D173Efs)];[0]	M; 6yr; urine cells	Sendai virus	qPCR; IF	EB differentiation	G-banding; Yes	Yes	[222]
*TRNT1*	RP	P1 (4 clones)	p.[(E43del)]; [(S418Vfs)]	M; 19 yr; Fib	Sendai virus	Morphology; RT-PCR; IF	Taqman mRNA scorecard	G-banding; N/D	N/D	[223]
*TRNT1*	RP	P2 (4 clones)	p.[(S418Kfs)]; c.[609-26T > C]	M; 21 yr; Fib	Sendai virus	Morphology; RT-PCR; IF	Taqman mRNA scorecard	G-banding; N/D	N/D	[223]
*TRNT1*	RP	P3 (4 clones)	p.[(S418Kfs)]; c.[609-26T > C]	M; 18 yr; Fib	Sendai virus	Morphology; RT-PCR; IF	Taqman mRNA scorecard	G-banding; N/D	N/D	[223]

Abbreviations: AGS, Aicardi-Goutières syndrome; AN, auditory neuropathy; AP, Alkaline phosphatase staining; CIV, complex IV; CM, cardiomyocyte; CMT, Charcot-Marie-Tooth disease; CoQ10, coenzyme Q10; COXPD, combined oxidative phosphorylation deficiency; DCMA, dilated cardiomyopathy with ataxia; DOA, dominant optic atrophy; EB, embryoid body; F, female; Fib, fibroblasts; Hep, hepatocyte; hypomethylation, hypomethylation of the OCT4 and NANOG promoter; HSP, hereditary spastic paraplegia; IF, Immunofluorescence; M, male; MSA, multiple system atrophy; mo, months; morphology, ES-like colony morphology; MSL, multiple symmetric lipomatosis; MTDPS, mtDNA depletion syndrome; N/D, no data; PBMC, peripheral blood mononuclear cells; PEO, progressive external ophthalmoplegia; RP, retinitis pigmentosa; wk, weeks; yr, year. ^a^ Unless provided in the original publication, consequences of mutations at the protein level were predicted based on the canonical transcript of the respective genes using the hg38 reference genome. Complicated mutations affecting splicing/intronic mutations are left in cDNA format. ^b^ Includes gender (if reported); age of patient (if reported); parental cell line. ^c^ Lineage validation comprises a range of genetic analysis techniques that permit DNA fingerprinting-based lineage tracing to the parental cell line. ^d^ Allele is listed according to original publication, due to ambiguity of the reported variant.

**Table A2:** mtDNA models.

Gene(s)	Disease	Cell Line ID	Mutation	Cell Line Origin ^a^	Reprogramming	Pluripotency	Trilineage Potential	Karyotype; Lineage Validation ^b^	Heteroplasmy Before (After) Reprogramming ^c^	Mycoplasma Check	Ref.
*MT-ATP6*	Leigh syndrome	iPSC (8993T/G) (8 clones)	m.8993T > G p.(L156R)	Fib	Sendai virus	Morphology; IF	Teratoma	G-banding; Yes	52% (32–87%)	Yes	[65]
*MT-ATP6*	Leigh syndrome	Leigh-iPSC (5 clones)	m.8993T > G p.(L156R)	Fib	Sendai virus	Morphology; IF	Teratoma	G-banding; Yes	100% (100%)	Yes	[65]
*MT-ATP6*	Leigh syndrome	LS1-hiPSC	m.8993T > G p.(L156R)	F; 3 yr; Fib	mRNA-miRNA combination	Morphology; RT-PCR; IF	Taqman mRNA scorecard; EB differentiation	N/D	85% (≤80%)	N/D	[171]
*MT-ATP6*	Leigh syndrome	L749.1	m.8993T > G p.(L156R)	M; 8 mo; Fib	Retrovirus	Morphology; AP; RT-PCR; IF; Hypomethylation	EB differentiation	G-banding; Yes	90% (N/D)	N/D	[224]
*MT-ATP6*	Leigh syndrome	GM13411 (3 clones)	m.8993T > G p.(L156R)	M; 8 mo; Fib	Retrovirus	RT-PCR; IF	N/D	G-banding; N/D	100% (100%)	N/D	[136]
*MT-ATP6*	Leigh syndrome	TFA1 (3 clones)	m.9185T > C p.(L220P)	F; 80 yr; Fib	Episomal vectors	N/D	Teratoma; EB differentiation	G-banding; Yes	100% (100%)	Yes	[98]
*MT-ATP6*	Leigh syndrome	TDA2.3	m.9185T > C p.(L220P)	F; 47 yr; Fib	Retrovirus	N/D	Teratoma; EB differentiation	G-banding; Yes	100% (100%)	Yes	[98]
*MT-ATP6*	Leigh syndrome	TDA3.1	m.9185T > C p.(L220P)	F; 20 yr; Fib	Retrovirus	N/D	Teratoma; EB differentiation	G-banding; Yes	100% (100%)	Yes	[98]
*MT-ND1*	LHON	FINCBi001-A	m.3460G > A p.(A52T)	F; 21 yr; Fib	Sendai virus	Morphology; AP; RT-PCR; IF	EB differentiation	CGH-array; Yes	100% (100%)	Yes	[225]
*MT-ND4*	LHON	LHON V31-1 (3 clones)	m.11778G > C p.(R340P) ^d^	M; 18 yr; Fib	Episomal vector	Morphology; IF	Teratoma; EB differentiation	N/D	100% (N/D)	N/D	[226]
*MT-ND4*	LHON	LHON T1-20 (3 clones)	m.11778G > C p.(R340P) ^d^	M; 33 yr; Fib	Episomal vector	Morphology; IF	Teratoma; EB differentiation	N/D	100% (N/D)	N/D	[226]
*MT-ND4*	LHON	TVGH-iPSC-010	m.11778G > A p.(R340H)	F; 61 yr; PBMC	Sendai virus	Morphology; RT-PCR; FACS	Teratoma; EB differentiation	G-banding; Yes	100% (N/D)	Yes	[227]
*MT-ND4*	LHON	LHON-affected	m.11778G > A p.(R340H)	PBMC	Sendai virus	AP; RT-PCR; IF	N/D	N/D	100% (>98%)	N/D	[165]
*MT-ND4*	LHON (unaffected)	LHON-carrier	m.11778G > A p.(R340H)	PBMC	Sendai virus	AP; RT-PCR; IF	N/D	N/D	100% (>98%)	N/D	[165]
*MT-ND4*	LHON	LHON-iPSC	m.11778G > A p.(R340H)	M; 18 yr; PBMC	Sendai virus	Morphology; RT-qPCR	N/D	N/D	N/D	N/D	[101]
*MT-ND6 and MT-ND1*	‘LHON plus’	LHON Q1-4 (3 clones)	m.14484T > C p.(M64V) and m.4160T > C p.(L285P)	F; 30 yr; Fib	Episomal vector	Morphology; IF	Teratoma; EB differentiation	N/D	100% (N/D)	N/D	[226]
*MT-ND5*	MELAS	M-iPS (2 clones)	m.13513G > A p.(D393N)	Fib	Lentivirus	Morphology; RT-qPCR; IF	EB differentiation	N/D; Yes	47% (50%)	N/D	[69]
*MT-ND5*	Leigh syndrome	iPSC (13513G/A) (5 clones)	m.13513G > Ap.(D393N)	Fib	Sendai virus	Morphology; IF	Teratoma	G-banding; Yes	84% (32–100%)	Yes	[65]
*MT-ND5*	Leigh syndrome	LND554SV.3	m.13513G > A p.(D393N)	M; Fib	Sendai virus	Morphology; AP; qPCR; IF; hypomethylation	EB differentiation	G-banding; Yes	55% (32%)	N/D	[228]
*MT-ND5*	MELAS	MELAS-iPSC A01 (2 clones)	m.13513G > A p.(D393N)	M; 16 yr; Fib	Episomal vectors	Morphology; RT-PCR; IF	EB differentiation	G-banding; N/D	20% (>60%)	Yes	[81]
*MT-RNR1*	SNHL	IBMSi004-A	m.1555A > G	F; 39 yr; PBMC	Sendai virus	RT-PCR; IF; FACS;	Teratoma; EB differentiation	G-banding; Yes	N/D (84.7%)	Yes	[229]
*MT-RNR2^e^*	HCM	HCM-iPSC	m.2336T > C ^e^	Urine cells	Retrovirus	AP; qPCR; IF; hypomethylation	Teratoma; EB differentiation	G-banding; N/D	100% (N/D)	Yes	[230]
*MT-TK*	MERRF	M1-iPSC	m.8344A > G	F; 15 yr; Fib	Retrovirus	Morphology; AP; RT-PCR; IF	Teratoma; EB differentiation	N/D	90% (70%)	N/D	[231]
*MT-TK*	MERRF	M2-iPSC	m.8344A > G	F; 13 yr; Fib	Retrovirus	Morphology; AP; RT-PCR; IF	Teratoma; EB differentiation	N/D	60% (60%)	N/D	[231]
*MT-TK*	MERRF	TVGH-iPSC-MRF-M^High^	m.8344A > G	F; 15 yr; Fib	Retrovirus	RT-PCR; IF	Teratoma; EB differentiation	G-banding; Yes	N/D (76%)	Yes	[232]
*MT-TL1*	MIDD	Mt1 (2 clones)	m.3243A > G	M; 38 yr; Fib	Retrovirus	Morphology; AP; IF; hypomethylation	Teratoma; EB differentiation	G-banding; Yes	18% (51% and 87%)	N/D	[233]
*MT-TL1*	MIDD and MELAS	Mt2 (4 clones)	m.3243A > G	F; 46 yr; Fib	Retrovirus	Morphology; AP; IF; hypomethylation	Teratoma; EB differentiation	G-banding; Yes	24% (69–83%)	N/D	[233]
*MT-TL1*	MIDD	MH1, MH2 and MH3 (3 clones)	m.3243A > G	M; 39 yr; Fib	Retrovirus	Morphology; IF; RT-PCR	Teratoma	G-banding; Yes	22% (>80%)	N/D	[21]
*MT-TL1*	CM	P3-iPSC	m.3243A > G	F; 55 yr; Fib	Retrovirus	N/D	N/D	N/D; Yes	35% (>80%)	N/D	[21]
*MT-TL1*	MELAS	MELAS-iPSC (5 clones)	m.3243A > G	Fib	Sendai virus	Morphology; IF	Teratoma	G-banding; Yes	29% (33–100%)	Yes	[65]
*MT-TL1*	MELAS	Patient #1- iPSCs	m.3243A > G	Fib	Episomal plasmid	Morphology; IF	EB differentiation	N/D	~99% (100%)	N/D	[90]
*MT-TL1*	MELAS	Patient #2- iPSCs (3 clones)	m.3243A > G	Fib	Episomal plasmid	Morphology; IF	EB differentiation	N/D	~69% (>70–100%)	N/D	[90]
*MT-TL1*	MELAS	Patient #3- iPSCs (3 clones)	m.3243A > G	Fib	Episomal plasmid	Morphology; IF	EB differentiation	N/D	~55% (60–100%)	N/D	[90]
*MT-TL1*	MELAS	MELAS-iPSC (16 clones)	m.3243A > G	Fib	Retrovirus	Morphology; IF	Teratoma; EB differentiation	G-banding; N/D	78% (40–99%)	N/D	[234]
*MT-TL1*	MELAS	MiPSC5	m.3243A > G	M; Fib	Sendai virus	Morphology; qPCR; IF	Teratoma	G-banding; Yes	90% (>80%)	N/D	[80]
*MT-TL1*	MELAS	MitoA hiPSCs (7 clones)	m.3243A > G	F; 17 yr; Fib	Sendai virus	N/D	N/D	G-banding; Yes	17% (47–82%)	N/D	[87]
*MT-TL1*	MELAS	MitoB hiPSCs (5 clones)	m.3243A > G	F; 79 yr; Fib	Sendai virus	N/D	N/D	G-banding; Yes	45% (38–81%)	N/D	[87]
*MT-TL1*	MELAS	MitoC hiPSCs (9 clones)	m.3243A > G	M; 31 yr; Fib	Sendai virus	N/D	N/D	G-banding; Yes	47% (38–83%)	N/D	[87]
*MT-TL1*	MELAS	HH1	m.3243A > G	M; 42 yr; Fib	Retrovirus	qPCR; IF	N/D	G-banding; N/D	85% (71%)	N/D	[97]
*MT-TL1*	MELAS	P1 iPSCs (2 clones)	m.3243A > G	F; 73 yr; Fib	Sendai virus	IF; FACS	Spontaneous monolayer differentiation	SNP-array; Yes	~70% (50% and 70% ^f^)	N/D	[235]
*MT-TW*	MELAS	Patient (3 clones)	m.5541C > T ^g^	M; <15 yr; Myoblast	Episomal plasmid	Morphology; IF	EB differentiation	N/D	~100% (~100%)	Yes	[46]
*MT-ND4/5; MT-TL2/TS2/TH*	Pearson syndrome	PS-iPS (3 clones)	m.10949_13449del (2501bp deletion)	F; 3 yr; Fib	Retrovirus	Morphology; AP; RT-PCR;IF	Teratoma	N/D	~70% (55–70%)	N/D	[45]
*MT-ATP6; MT-CO3;* *MT-ND3/4/4L/5/6; MT-CYB;* *MT-TG/TR/TL2/* *TS2/TH/TE*	Pearson syndrome	GM04516PS-iPSC	m.8824_15854del (7031bp deletion)	F; 5 yr; Fib	Retrovirus	AP; IF	Teratoma	N/D	6% (20%)	N/D	[45]
*MT-CO3; MT-ND3/4/4L/5/6*	Pearson syndrome	FINCBi002-A	m.9449_14550del (5102bp deletion)	M; 5 mo; Fib	Sendai virus	Morphology; AP; RT-PCR; IF	EB differentiation	Microarray; Yes	50% (80%)	Yes	[236]
*MT-ATP6; MT-CO3; MT-ND3/4/4L/5*	Pearson syndrome	FINCBi003-A	m.8469_13460del (4992bp deletion)	M; 8 yr; Fib	Sendai virus	Morphology; AP; RT-PCR; IF	EB differentiation	Microarray; Yes	10% (30%)	Yes	[236]

Abbreviation: AP, Alkaline phosphatase staining; CM, cardiomyopathy; EB, embryoid body; F, female; Fib, fibroblasts; HCM, hypertrophic cardiomyopathy; hypomethylation, hypomethylation of the OCT4 and NANOG promoter; IF, Immunofluorescence; LHON, Leber’s hereditary optic neuropathy; M, male; MELAS, mitochondrial encephalopathy, lactic acidosis and stroke-like episodes; MERRF, myoclonus epilepsy associated with ragged red fibres; MIDD, maternally inherited diabetes and deafness; mo, months; morphology, ES-like colony morphology; N/D, no data; PBMC, peripheral blood mononuclear cells; SNHL, sensorineural hearing loss; wk, weeks; yr, years. ^a^ Includes gender of patient; age of patient (if reported); parental cell line. ^b^ Lineage validation comprises a range of genetic analysis techniques that permit DNA fingerprinting-based lineage tracing to the parental cell line. ^c^ Cell lines and clones with low/no mutant load (<30%) following reprogramming have been excluded. Clones that were generated during reprogramming, but no follow-up characterisation was performed have also been excluded. ^d^ The DNA chromatogram presented in the publication indicates that this variant is actually the common LHON m.11778G > A [p.(R340H)] variant, rather than the indicated m.11778G > C. ^e^ The reported m.2336T > C variant is not yet confirmed as pathogenic and *MT-RNR2* is not yet known as a mitochondrial disease gene. However, the variant is absent from population databases (MITOMAP and gnomAD v3.1) and the hPSC studies provide support for pathogenicity. ^f^ Clone with 70% mutant heteroplasmy had abnormal karyotype. ^g^ The m.5541C > T variant is not yet confirmed as pathogenic, although in silico predictions indicate it is “likely pathogenic” (MITOMAP) and it is absent from population databases (MITOMAP and gnomAD v3.1).

**Table A3:** Summary of functional defects of hPSC-derived clinically relevant cell types of nDNA associated mitochondrial diseases.

Disease (Gene)	Cell Line ID	Genetic Mutation	Isogenic Controls ^a^	Cell Type	OXPHOS Defects ^b^	Other Mitochondrial Defects ^b^	Cellular and Physiological Defects ^b^	In Vitro Therapeutic Studies	Ref.
HSP *(ATAD3A)*	HEL142	p.[(G355D)];[=]	−	Neurons	N/D	Mitochondrial network abnormalities	↑ lysosome accumulation	N/D	[196]
MTDPS3 (*DGUOK*)	DGUOK^Δ14/Δ5^ iPSC	p.[W166*]; [H167Lfs*213]	+ +	Hep, Hep- org.	↓ maximal and basal OCR (3/3) ↓ ATP ^c^ ↓ MMP (3/3) ↑ lactate levels (3/3)	Abnormal mitochondrial ultrastructure (1/3) ↑ ROS levels (3/3) ↓ mtDNA depletion (3/3)	↑ susceptibility to iron overload- induced ferroptosis (2/2)NCOA4-dependent ferritin degradation in lysosomes (2/2)	Drug screen on library of 2400 drugs; NAD treatment restored mitochondrial bioenergetics and improved expression of ETC genes. DFO, Fer-1 (ferroptosis inhibitors) and NAC rescued iron-overload induced ferroptosis.	[131,132]
	Patient 1	p.[F256*];[F256 *]	+ −						
	Patient 2	[p.A2S; c.591G>A]; [c.142+1G>A],	+ −						
DCMA *(DNAJC19)*	Patient 1	c.[130-1G>C]; [130-1G>C]	−	CM	N/D	Fragmented mitochondrial network (2/2)	↓ L-OPA1 isoform expression (2/2)	Elamipretide improved mitochondrial fragmentation and restored OPA1 isoform balance	[201]
	Patient 2	c.[130-1G>C]; [130-1G>C]	−						
CMT2A *(MFN2)*	Patient 1 (2 clones)	p.[(R364W)];[=]	− −	SpMN	N/D	Impaired mitochondrial motility (2/2) Clustering of mitochondria around nucleus (1/1) ↑ mitophagy; ↓ mtDNA content (1/1)	Aberrant electrophysiological activity (hyperpolarised action potential, increased sodium channel density) (1/1) Altered gene expression (↑ OXPHOS; ↓ neurogenesis) (1/1) ↓ sensitivity to apoptosis (1/1)	N/D	[191,207]
	CMT2A-1/2	p.[(A383V)];[=]	− −						
MSA-C *(COQ2)*	MSA_A (3 clones)	p.[(R387*)]; [(V393A)]	+ + + − −	Neurons	↓ maximal and basal OCR ↓ ATP ^d^	↓ CoQ10 (and vitamin E) levels ↑ ROS levels	↑ apoptosis (in galactose-based media)	N/D	[198]
CoQ10 deficiency *(COQ4)*	CQ4-iPSC	p.[(E161D)];[=]	+ −	SkMC	↓ Complex I + III activity	↑ ROS levels Mitochondrial morphology defects	Differentiation defect ^e^	N/D	[200]
DOA and Parkinson’s disease *(OPA1)*	VO-iPSC	c.[(2496+1G>T)];[=]	−	NSC, NPC, RGC, and/or Neurons	↓ maximal and basal OCR (4/5) ↓ ATP ^c,d^ (4/5) ↑ glycolysis (2/2) ↓ MMP (2/2) CI defect (↓ CI subunit expression, activity and/or CI related ATP synthesis) (4/4)	Fragmented mitochondrial network (4/5) ↑ ROS levels (3/3)	Differentiation defects ^e^ and/or increased neurodegeneration over prolonged culture (7/7) Reduced mitochondrial mass and loss of active synaptic terminals throughout prolonged culture (microfluidic neuronal Nigro-striatal pathway model) (1/1)	NAC, Z-VAD (apoptosis inhibitor), and Nec-1 (necrosis inhibitor) improved survival of neurons derived from NPCs.	[104,119,120,123,211]
	OL-iPSC	c.[(2496+1G>T)];[=]	−						
	PD-OPA1 G488R (2 clones)	p.[G488R];[=]	+ −						
	PD-OPA1 A495V #72	p.[A495V[;[=]	+ −						
	Opa1P (2 clones)	c.[(33-34ins9)];[=]	− −						
	Opa1 (2 clones)	c.[(33-34ins9)];[=]	− −						
	OPA1+/− hESC	N/D (haploinsufficiency)	+						
CMTX6 *(PDK3)*	iPSCCMTX6	p.[(R158H)];[0]	+	SpMN	↓ ATP ^c,d^	Fragmented mitochondrial network Mitochondrial trafficking defect	PDC E1 hyperphosphorylation	Treatment with the pan PDK inhibitor DCA reduced PDC E1 hyperphosphorylation, and improved mitochondrial fragmentation and motility.	[216]
Leigh-like syndrome *(PMPCB)*	DII-2 iPSC (3 clones)	p.[I422T];[I422T]	− − −	NESC	N/D	N/D	Inefficient processing and accumulation of intermediate Frataxin; increased ISD complex formation; increased Fe-S cluster biogenesis	N/D	[217]
Autosomal dominant POE and parkinsonism *(POLG)*	CSC-35 (3 clones)	p.[(Q811R)];[=]	−	MDNS	↑ glycolysis	N/D	Accumulation of oligomeric αSYN Altered gene expression ↑ pigmentation in dopaminergic neurons	N/D	[190]
Autosomal recessive POE *(POLG)*	WS5A (3 clones)	p.[W748S]; [W748S]	− − −	NSC, and/or Neurons	↓ ATP ^c^ (2/2) CI defect (↓ CI expression) (2/2)	↑ ROS levels (2/2) mtDNA depletion (2/2)	N/D	NAC treatment on neurons reduced ROS levels and improved MMP.	[125,126]
	CP2A (2 clones)	p.[A467T]; [W748S]	− − −						
Alpers syndrome *(POLG)*	AHS iPS 1	p.[A467T];c.[1251-2A>T]	− −	Hep	↓ ATP ^c^ (2/2)	↑ frequency of mPTP opening and superoxide release (2/2) Abnormal mitochondrial ultrastructure (2/2) ↑ numbers of mitochondria without mtDNA (2/2)	↑ VA induced apoptosis (2/2)	CsA (mPTP specific inhibitor) reduced VA induced apoptosis. MnTMPyP and TEMPO (antioxidants) no effect. NAC and carnitine reduced VA induced apoptosis.	[129]
	AHS iPS 2	p.[A467T];c.[3626_3629dup]	− −						
MTDPS8A/B *(RRM2B)*	RRM2B^−/−^ iPSC	p.[(R36Sfs*55)]; [(R36Sfs*55)]	+	Hep	↓ ATP ^c^	mtDNA depletion	N/D	NAD treatment improved ATP levels.	[131]
CIV deficiency *(SCO2)*	SCO2G193S	p.[(G193S)]; [(G193S)]	− − −	CM	N/D	Abnormal mitochondrial ultrastructure (1/2)	Calcium homeostasis defects (2/2) Contractile defects (2/2)	Poor/no response to ionotropic agents.	[137]
	SCO2E140K	p.[(E140K)]; c.[(17ins(19))] ^f^	− − −						
Leigh syndrome *(SURF1)*	SURF1_Mut: S1	p.[(V177G)]; [(V177G)]	− −	NPC, Neurons, and/or Neural-org.	↓ CIV activity (3/3) ↓ maximal and basal OCR (2/2) ↑ lactate levels (2/2) ↑ glycolysis (1/1)	N/D	Differentiation defects ^e^ (3/3) ↓ neurite length and branching (3/3) Aberrant action potentials kinetics (3/3) ↑ proliferation and pluripotency markers (1/1) Disrupted WNT, TGFß, and SHH signalling (1/1)	Antioxidants (NAC and AT3), glucose and pyruvate supplementation showed minimal improvement in NPC and neurons. Hypoxia negatively impacted neurite length and branching defects. 400uM bezafibrate treatment alleviated OXPHOS and maturation defects in iPSC-NPCs.	[99]
	SURF1_Mut: S2	p.[(G257R)]; [(G257R)]	+ +						
	C1_Mut (2 clones)	p.[(G257R)]; [(G257R)]	+ +						
Barth syndrome *(TAFAZZIN)*	TAZ10	p.[G197V];[0]	-	CM, Cardiac-org.	↑ basal OCR (compensatory mechanism) (5/5) ↓ maximal OCR (5/5) ↓ ATP ^c^ in galactose culture (4/4) ↓ CII expression (1/1)	↑ ROS levels (2/2) Cardiolipin remodelling defects (4/4) Mitochondrial network fragmentation (1/1) Disrupted OXPHOS supercomplex assembly (1/1)	Disrupted sarcomeric organization at single cell level (4/5) Contractile defects (4/4) Metabolic alterations and substrate utilization (1/1) Abnormal calcium handling (2/2); Increased diastolic calcium leak through RYR2 (1/1) ↓ HIF1α signalling under hypoxic conditions (1/1)	BL corrected cardiolipin ratio. Arg + Cys supplementation improved ATP levels. LA improved MLCL:CL ratio, increased ATP levels, restored basal OCR. LA and MitoTEMPO reduced ROS, and improved sarcomere organisation and contractile defects.	[103,109,110,113,237]
	BTH-H	p.[(D173Tfs*12)];[0]	+ − − −						
	BTH-C	p.[(S110P)];[0]	− − −						
	PGP1-TAZ ^c.517delG^	p.[(D173Tfs*12)];[0]	+						
	PGP1-TAZ ^c.517ins^	p.[(D173Efs)];[0]	+						
RP *(TRNT1)*	P1	p.[(E43del)]; [(S418Vfs)]	− − − − − − −	Retinal-org.	N/D	↑ oxidative stress (3/3)	↑ autophagy (accumulation of LC3-II; decreased LAMP1 expression) (3/3)	N/D	[223]
	P2	p.[(S418Kfs)]; c.[609-26T>C]	− − − − − − −						
	P3	p.[(S418Kfs)]; c.[609-26T>C]	− − − − − − −						

Abbreviations: αSYN, alpha-Synuclein; AT3, α-Tocotrienol; BL, Bromoenol lactone; Cardiac-org, cardiac organoid; CI-CIV, OXPHOS complexes I-IV; CM, cardiomyocytes; CMT, Charcot-Marie-Tooth disease; CoQ10, coenzyme Q10; CsA, Cyclosporin A; DCA, dichloroacetate; DCMA, dilated cardiomyopathy with ataxia; DFO, deferoxamine; DOA, dominant optic atrophy; Fer-1, ferrostatin-1; FGF2, basic fibroblast growth factor; Hep, hepatocyte-like cells; Hep-org; hepatocyte organoid; HSP, hereditary spastic paraplegia; LA, linoleic acid; MDNS, midbrain dopaminergic neural spheroid; MMP, mitochondrial membrane potential; mPTP, mitochondrial permeability transition pore; MSA, multiple system atrophy; MTDPS, mtDNA depletion syndrome; NAC, N-acetylcysteine; NAD, nicotinamide adenine dinucleotide; NESC, neuroepithelial stem cell; Neural-org, neural organoid; NPC, neural progenitor cells; NSC, neural stem cells; N/D, no data; OCR, oxygen consumption rate; PDC, pyruvate dehydrogenase complex; POE, progressive external ophthalmoplegia; Retinal-org, retinal organoid; RGC, retinal ganglion cells; ROS, reactive oxygen species; RP, retinitis pigmentosa; RYR2, ryanodine receptor 2; SkMC, skeletal muscle cells; SpMN, spinal cord motor neurons; VA, valproic acid; +, one isogenic control; −, one non-isogenic control; ↑, increased; ↓, decreased. ^a^ Refer to specified publication for the combinations of controls, or number of clones per control line used for each assay. ^b^ Numbers in parentheses indicate the number of separate hPSC cell lines showing this response versus those tested. ^c^ Steady-state total cellular ATP. ^d^ ATP-linked respiration. ^e^ Differentiation defects correspond to either a reduced differentiation efficiency, or reduced maturity of cells following differentiation compared to controls. ^f^ Allele is listed according to original publication, due to ambiguity of the reported variant.

**Table A4:** Functional defects of hPSC-derived clinically relevant cell types of mtDNA associated mitochondrial diseases.

Disease (Gene)	Cell Line ID	Genetic Mutation ^a^	Isogenic Controls ^b^	Cell Type	OXPHOS Defects ^c^	Other Mitochondrial Defects ^c^	Cellular and Physiological Defects ^c^	In Vitro Therapeutic Studies	Ref.
Leigh syndrome *(MT-ATP6)*	TFA1	m.9185T > C (100%; p.L220P)	− − − − −	NPC, Neurons	↓ ATP ^d,f^ (4/4) ↑ MMP (4/4) ↓ basal OCR (1/1)	↑ ROS (1/1)	Calcium homeostasis defect (↓ calcium-induced calcium release; (↓ mitochondrial calcium release) (3/3) ↑ neurodegeneration (1/1)	Tested 130 FDA approved drugs; Avanafil (PDE5 inhibitor) partially rescued calcium homeostasis defect in NPCs and neuronsRapamycin treatment improved ATP production, decreased aberrant AMPK activation, and decreased glutamate induced toxicity	[98,136]
	TDA2.3	m.9185T > C (100%; p.L220P)	− − − − −						
	TDA3.1	m.9185T > C (100%; p.L220P)	− − − − −						
	GM13411(3 clones)	m.8993T > G (100%; p.L156R)	− −						
Leigh syndrome *(MT-ND5)*	LND554SV.3	m.13513G > A (19%; p.D393N)	−	NSC, Neurons	↓ basal and maximal OCR	N/D	↑ cell death Calcium homeostasis defect (↓ calcium buffering capacity)	Treatment with the succinate prodrug NV241 improved mitochondrial respiration; however, similar observations were detected from DMSO control	[102]
Leigh syndrome *(MT-ATP6)*	Leigh iPSC	m.8993T > G (100% ^i^; p.L156R)	+	SkMC	↓ ATP ^e^	N/D	N/D	N/D	[65]
LHON *(MT-ND4)*	LHON-affected	m.11778G > A (98.25%; p.R340H)	− −	RGC	↓ basal OCR (2/2)	↑ mtDNA copy number ↑ ROS (2/2)	Differentiation defect ^g^ (2/3) Aberrant electrophysiological activity (↓ action potential peaks) (1/1) Defect in glutamate uptake (1/1) ↑ apoptosis (1/2) Changes in mitochondrial transport; reduced KIF5A expression (1/2)	NAC reduced ROS levels and apoptosis, and restored KIF5A expression and mitochondrial motility	[101,165,166]
	LHON-unaffected	m.11778G > A (98.42%; p.R340H)	−						
	LHON-iPSC	m.11778G > A (N/D; p.R340H)	−						
LHON *(MT-ND1* and *MT-ND6)*	LHON Q1-4 (3 clones)	m.4160T > C (p.L285P)/ m.14484T > C (p.M64V) (100% ^i^)	+ −	RGC		↑ ROS	↑ apoptosis	N/D	[64]
HCM (*MT-RNR2*) ^h^	HCM-iPSC	m.2336T > C (N/D)	− − −	CM	↓ MMP	Mitochondrial ultrastructural defects ↑ mtDNA copy number ↓ stability of *16s rRNA*	Calcium homeostasis defects Aberrant electrophysiological activity	N/D	[230]
MERRF *(MT-TK)*	M1-iPSC	m.8344A > G (≥40%)	+	CM, NPC	↓ basal and maximal OCR (2/2) ↓ ATP ^e^ (2/2)	↑ ROS (2/2) Mitochondrial fragmentation/morphology defects (2/2)	↑ expression of antioxidant genes catalase and CuZnSOD (2/2)	N/D	[231]
	M2-iPSC	m.8344A > G (>40%)	−						
MELAS *(MT-TL1)*	P1 iPSCs (2 clones ^j^)	m.3243A > G (>55%)	− − −	RPE	N/D	Features of mitochondrial fragmentation/morphology defects	Atypical spatial distribution of RPE Underdeveloped microvilliAberrant melanosome morphology ↓ phagocytosis of POS	N/D	[235]
MELAS *(MT-TL1)*	MiPSC5	m.3243A > G (~80%)	+ −	EC	N/D	↑ mitochondrial biogenesis ↑ ROS	Differentiation defects ^g^ ↓ cell migration and tube formation ↑ apoptosis ↑ uptake and oxidation of LDL ↑ expression of VCAM-1 isoform b ↑ monocyte adhesion to EC	Edaravone (anti-oxidant), CoQ10 and Vit C improved endothelial tube formation and reduced ROS levels. Edaravone also reduced basal inflammation	[48]
MIDD and MELAS *(MT-TL1)*	HH1	m.3243A > G (>60%)	+ −	Neurons, NPC, Spinal- organoid	↓ basal and maximal OCR (3/3) ↓ ATP ^e^ (3/3) ↑ glycolysis (1/1) CI deficiency (1/1)	↓ mitochondrial content along axons (1/1) Changes in mitochondrial translation (1/1)	Differentiation defects^g^ (1/1) Structural/morphological defects (2/2) ↓ synaptic density (1/1) ↓ frequency of spontaneous excitatory activity at single cell level (1/1) ↓ spontaneous MFR and NBR at a network level (3/3) ↑ PRS outside network bursts (3/3) Selective accumulation of CI in autophagosomes and clearance by mitophagy upon neuronal differentiation (1/1) Hyperactive Notch signalling (1/1)	DAPT (Notch signalling inhibitor) improved neurodevelopmental defects (improved motor neuron differentiation efficiency and spinal organoid neurite outgrowth defects)	[21,65,80,97,168]
	MitoA hiPSCs (HH2)	m.3243A > G (>60% ^i^)	+ −						
	MitoB hiPSCs (HH3)	m.3243A > G (65%)	+ −						
	MELAS-iPSC (2 clones)	m.3243A > G (100% ^h^)	+						
	MH1, MH2 and MH3 (3 clones)	m.3243A > G (>80%)	+ − −						
	MiPSC5	m.3243A > G (80%)	+ −						
Pearson syndrome (2501bp macro-deletion)	PS-iPS	m.10949_13449del (50% ^i^)	+ +	HPC	N/D	N/D	Accumulation of iron granules in erythroid precursors (i.e., increased formation of sideroblasts)	N/D	[45]

Abbreviations: AMPK, AMP-activated protein kinase; CM, cardiomyocytes; DMSO, dimethyl sulfoxide; EC, endothelial cells; HCM, hypertrophic cardiomyopathy; HPC, hematopoietic progenitor cells; KIF5A, kinesin family member 5A protein; LDL, low-density lipoprotein; MELAS, mitochondrial encephalopathy, lactic acidosis and stroke-like episodes; MERRF, myoclonus epilepsy associated with ragged red fibres; MFR, mean firing rate; MIDD, maternally inherited diabetes and deafness; MMP, mitochondrial membrane potential; NAC, N-acetylcysteine; NBR, network burst rate; NPC, neural progenitor cells; NSC, neural stem cells; OCR, oxygen consumption rate; PDE5; phosphodiesterase type 5; POS, photoreceptor outer segment; PRS, percentage of random spikes; RGC, retinal ganglion cells; ROS, reactive oxygen species; RPE, retinal pigment epithelium cells; SCNT, somatic cell nuclear transfer; SkMC, skeletal muscle cells; Vit C, Vitamin C; ↑, increased; ↓, decreased. ^a^ mtDNA heteroplasmy level in differentiated hESC/iPSC-derived cell type (if reported) and predicted protein outcome. ^b^ Refer to specified publication for the combinations of controls, or number of clones per control line used for each assay. +, one isogenic control; −, one non-isogenic control ^c^ Numbers in parentheses indicate the number of separate hPSC cell lines showing this response versus those tested. ^d^ Steady-state total cellular ATP. ^e^ ATP-linked respiration. ^f^ ATP synthesis over time. ^g^ Differentiation defects corresponds to either a reduced differentiation efficiency or reduced maturity of cells following differentiation compared to controls. ^h^ *MT-RNR2* is not yet known as a mitochondrial disease gene and the m.2336T > C variant is not yet confirmed as pathogenic. However, the variant is absent from population databases (MITOMAP and gnomAD v3.1) and the hPSC studies provide support for pathogenicity. ^i^ mtDNA heteroplasmy level reported in undifferentiated hESC/iPSC only. ^j^ One of the clones used in this study had an abnormal karyotype.
